# Global heart warming: kama muta evoked by climate change messages is associated with intentions to mitigate climate change

**DOI:** 10.3389/fpsyg.2023.1112910

**Published:** 2023-04-28

**Authors:** Beate Seibt, Janis H. Zickfeld, Nora Østby

**Affiliations:** ^1^Department of Psychology, University of Oslo, Oslo, Norway; ^2^Centro de Investigação e Intervenção Social (CIS-IUL), Instituto Universitário de Lisboa (ISCTE-IUL), Lisboa, Portugal; ^3^Department of Management, Aarhus University, Aarhus, Denmark; ^4^Institutt for Psykologi, Pedagogikk og Juss, Kristiania University College, Oslo, Norway

**Keywords:** climate change, intentions, tears, emotions, media effects, being moved, environmental psychology, sustainability

## Abstract

Concern about climate change is often rooted in sympathy, compassion, and care for nature, living beings, and future generations. Feeling sympathy for others temporarily forms a bond between them and us: we focus on what we have in common and feel a sense of common destiny. Thus, we temporarily experience *communal sharing relationships*. A sudden intensification in communal sharing evokes an emotion termed *kama muta*, which may be felt through tearing up, a warm feeling in the chest, or goosebumps. We conducted four pre-registered studies (*n* = 1,049) to test the relationship between kama muta and pro-environmental attitudes, intentions, and behavior. In each study, participants first reported their attitudes about climate change. Then, they received climate change-related messages. In Study 1, they saw one of the two moving video clips about environmental concerns. In Study 2, participants listened to a more or less moving version of a story about a typhoon in the Philippines. In Study 3, they listened to a different, also moving version of this story or an unrelated talk. In Study 4, they watched either a factual or a moving video about climate change. Participants then indicated their emotional responses. Finally, they indicated their intentions for climate mitigation actions. In addition, we measured time spent reading about climate-related information (Studies 1, 2, and 4) and donating money (Study 4). Across all studies, we found that feelings of kama muta correlated positively with pro-environmental intentions (*r* = 0.48 [0.34, 0.62]) and behavior (*r* = 0.10 [0.0004, 0.20]). However, we did not obtain evidence for an experimental effect of the type of message (moving or neutral) on pro-environmental intentions (*d* = 0.04 [−0.09, 0.18]), though this relationship was significantly mediated by felt kama muta across Studies 2–4. The relationship was not moderated by prior climate attitudes, which had a main effect on intentions. We also found an indirect effect of condition through kama muta on donation behavior. In sum, our results contribute to the question of whether kama muta evoked by climate-change messages can be a motivating force in efforts at climate-change mitigation.

## Introduction

Extreme weather events such as storms and floods have increased globally due to climate change, and they will further increase. However, we still have a chance to limit these impacts to a level that preserves the achieved global human wellbeing throughout the 21st century (Hoegh-Guldberg et al., 2018, p. 279). To reach such a best-case scenario, however, global warming needs to be limited to 1.5°C, which requires “rapid, far-reaching and unprecedented changes in all aspects of society” (Allen et al., 2018). People must implement these changes. Thus, a new consensus on the urgency of measures to mitigate climate change needs to emerge. What, then, will make people feel the urgency and increase their willingness to act on it? And what role do their emotional responses to climate change messages play in feeling this urgency? We propose here that a particular emotional response to climate change messages, that of being moved or *kama muta*, contributes to the willingness to act. We will first briefly review how media content can provoke emotions that motivate climate action and then derive our hypotheses on kama muta as a motivating force. We then present an overview of the four studies we conducted to test these assumptions.

### Negative emotions evoked by media can motivate climate action

Information on climate change and effective climate actions is a prerequisite for wanting to mitigate climate change. Learning about environmental issues thus led to more concern and a sense of responsibility toward the environment (Bradley et al., 1999; McMillan et al., 2004). Furthermore, articles from the New York Times persuaded college students to change their attitudes and to act on climate change, and students who were more open to change and to think deeply about issues were most willing to act (Sinatra et al., 2012).

However, information typically is not enough to motivate individuals to engage in climate actions (Abrahamse et al., 2005). For example, media coverage in Germany on the UN Climate Summit did not motivate Germans to engage in climate action (Brüggemann et al., 2017). Instead, emotions lend urgency to information (Tomkins, 1962), and thus, emotional responses to climate change messages can be expected to predict a message’s motivational impact.

Climate change worsens our collective prospects and evokes largely negative imagery (Lorenzoni et al., 2006). Thus, worry and anxiety are normal emotional responses to acknowledging the reality of climate change. The adaptive function of these emotional responses is to motivate preventive action (Mathews, 1990). Accordingly, individuals were more prone to engage in mitigation actions when they habitually worried about climate change (Verplanken and Roy, 2013). Similarly, in a survey among US-Americans on support for national climate policies, worry about climate change was the strongest emotional predictor (Smith and Leiserowitz, 2014).

If negative emotions about climate change predict climate action, then inducing such negative emotions through media messages seems a good strategy to promote climate action. Indeed, sadness-evoking videos led to more time spent with climate footprint calculators and more donations than neutral videos, but only as long as the sadness persisted (Schwartz and Loewenstein, 2017). Similarly, messages evoking fear can be effective, particularly when they are strong and when they are combined with suggestions for climate actions participants can easily do to reduce the risk (Witte and Allen, 2000).

Given that fear-inducing messages can be effective, it is plausible that messages about the consequences of climate change for oneself and one’s community are particularly effective. Accordingly, a local rather than a global message frame increased willingness to act in several studies (Lorenzoni et al., 2007; Spence et al., 2012; Scannell and Gifford, 2013; Evans et al., 2014; Reser et al., 2014). Similarly, personal experience with floods was associated with more willingness to act (Spence et al., 2011).

Taking responsibility for one’s contribution to climate change can also elicit guilt, namely when focusing on others’ worsening prospects (Baumeister et al., 1994). Guilt has also been found to predict climate action. Swiss students’ intention to save energy was best predicted by a composite index of guilt for wasting energy (called *personal norms*), among values, norms, attitudes, beliefs, control, appraisal, and emotion variables (Brosch et al., 2014).

The literature reviewed so far suggests that worry, fear, sadness, and guilt evoked by messages can motivate climate action. However, many studies evoking these emotions failed to find an effect on environmental intentions. For example, if the person does not feel responsible and able to do something about climate change, inducing fear can also lead to more defensiveness, i.e., to avoiding the issue (Brügger et al., 2015). Likewise, fear-inducing images representing climate change tend to inhibit action when they lead to feeling helpless and overwhelmed (O’Neill and Nicholson-Cole, 2009). Negative emotions about climate change, then, may be a strong motivator, but may not suffice to initiate and sustain climate action.

### What about positive emotions evoked by media?

There is mounting evidence to suggest that positive emotions motivate acting pro-environmentally (for an overview, see Schneider et al., 2021). While fear and worry lend urgency to messages, positive emotions such as hope and interest (Smith and Leiserowitz, 2014) may be more closely related to self-efficacy beliefs and positive action–outcome expectancies, thus making action intentions more likely (see Ajzen, 1991). Accordingly, a video showing landscape degradation after showing natural beauty evoked more environmental concern in climate skeptics than beauty or degradation alone (Franzen and Mader, 2020). For climate change skeptics, messages highlighting positive co-benefits of climate solutions are more effective than highlighting climate mitigation as the goal (Bain et al., 2012). In general, expecting to derive positive feelings from climate action motivates such action (Pelletier et al., 1998; Taufik et al., 2016; Schneider et al., 2017; van der Linden, 2018; Jia and van der Linden, 2020).

Some positive emotions, called self-transcendent emotions (Stellar et al., 2017), can direct a person’s focus away from short-term, selfish interests. Given that they may enable people to forgo some immediate benefits for the sake of larger communities’ benefits, they have the potential to motivate climate action. Awe, an emotional response to something vastly larger than oneself (Keltner and Haidt, 2003; Shiota et al., 2007), and elevation, an emotional response to witnessing moral excellence (Algoe and Haidt, 2009), are two self-transcendent emotions that have been examined in relation to climate change messages.

Awe about natural and social events that were unconnected to climate change increased the likelihood of engaging in environmental behaviors in Chinese college students *via* higher connectedness to nature (Yang et al., 2018) or lower social dominance orientation (Zhao et al., 2018). Comparing the effects of neutral, amusing, and elevating videos showing altruism, Moreton et al. (2019, Study 2) found no direct, but indirect effects of the elevation video condition on increased willingness to sacrifice for the environment and on intentions to engage in 12 environmental behaviors through a composite of self-transcendent emotions and connectedness to nature. The composite of the self-transcendent emotions, such as inspiration, love, feeling moved, respect, admiration, awe, gratitude, and humility, correlated 0.60 with connectedness to nature, 0.35 with intentions, and 0.26 with willingness to sacrifice. Thus, the elevation videos increased connectedness to nature despite not being about the natural environment, and to the extent that participants responded emotionally and felt more connected to nature after watching the video, and they were also moved to protect the environment.

Similarly, another study found indirect, but no direct, effects of viewing short videos on willingness to sacrifice to protect the environment and on donations to The Nature Conservancy (Diessner et al., 2022). A video on the beauty of planet Earth, compared with a video on flute making or a video showing rocks in the desert, promoted willingness and donations through elevation. A video depicting an altruistic Thai man promoted willingness and donations through awe and elevation, in combination but not individually, compared with the control videos. The level of the visual beauty of the videos positively predicted willingness and donations.

In sum, self-transcendent emotions may indeed play an important role in motivating climate action. However, the reviewed studies did not focus on evoking self-transcendent emotions *about climate change*. Thus, there is a need to investigate self-transcendent emotions induced by climate change messages, which do not deny the saddening and worrying aspects of climate change. Furthermore, awe and elevation are self-transcendent but not clearly relational according to their definitions. We wanted to study an emotion that is felt when relating to others. Kama muta is such an emotion, and there is reason to believe that it can motivate climate action.

### Kama muta as a motivator

Care, compassion, and love are relational, self-transcendent emotions that have been studied in the context of climate change mitigation. Care for those severely affected by climate change, which can be related to compassion, sympathy, or guilt, can be a stronger motivator than self-interest (Brügger et al., 2015; McDonald et al., 2015; Dickinson et al., 2016). Furthermore, examining the impact of the frequency with which participants experience various positive emotions in their lives, a series of studies found that only the composite index of the self-transcendent emotions of love, compassion, and awe, but not the self-interested emotions of joy, contentment, pride, and amusement, predicted climate action frequency and pro-environmental attitudes (Jacobs and McConnell, 2022; see also Jacobson et al., 2019; Zelenski and Desrochers, 2021).

For environmental causes, compassion, empathy, and sympathy are often directed at nature, places, ecosystems, “Mother Earth” and non-human living creatures (Schultz, 2002; Kashima et al., 2014; Pihkala, 2022), people hit by catastrophes (Schultz, 2001), or future generations (Brosch et al., 2014). Feeling compassionately with other beings temporarily forms a bond between them and us: we focus on what we have in common with others and feel a sense of common destiny. Thus, we temporarily experience *communal sharing (CS) relationships* with strangers or an intensification of existing communal sharing relationships, characterized by caring for each other according to need and ability and marked by touch, synchrony, and eating together, feeling unified, close, and connected (Fiske, 1992, 2004).

A sudden intensification in communal sharing evokes an emotion termed *kama muta*, Sanskrit for ‘moved by love’, which may be felt through tearing up, a feeling of warmth in the center of the chest, goosebumps, or a buoyant feeling right afterward (for an overview, see Fiske et al., 2019). Kama muta is defined and measured as the cooccurrence of an appraisal of sudden intensification of communal sharing, labeling the state as being moved or touched, labeling the elicitor as heartwarming, experiencing several of the bodily sensations, experiencing the feeling as positive, and increased care and commitment to communal relationships (Zickfeld et al., 2019). Across 16 studies and 2,918 participants, Zickfeld et al. (2017) found a correlation of 0.35 between empathic concern, the trait associated with responding with compassion and tender feelings to the suffering of other beings, and state kama muta, evoked by videos and autobiographical recollections. They characterized compassion as a subset of kama muta evoked by another’s need.

We, therefore, expect kama muta, evoked by love, connection, and compassion for other living beings, present and future, to increase willingness to act on climate change. People can also feel kama muta toward larger entities such as particular places, “the land,” “Mother Earth,” ecosystems, nature, or the oceans (Fiske, 2019). Consonant with this, showing images of distressed foxes (but not raccoons) increased compassion and wildlife conservation efforts (Greving and Kimmerle, 2021). Measuring dispositional compassion and inducing compassion for fellow humans through pictures unrelated to climate change, Pfattheicher et al. (2016) found compassion to predict pro-environmental intentions. Using a similar manipulation of compassion *via* instructions to empathize versus stay objective, Lu and Schuldt (2016) studied the effect of compassion on a victim of a drought described in a fictitious news article. They found increased support for climate policies in the compassion condition, particularly for conservatives, and mediated *via* compassion and belief in anthropogenic drought. These findings suggest that kama muta evoked by feeling closer to others in need indeed increases the motivation to mitigate climate change.

What about kama muta more generally? Are there other ways in which climate change messages can lead to increased kama muta? Political ads for election campaigns were found to evoke kama muta particularly in partisans, which in turn increased motivation to support the candidate (Seibt et al., 2019; Grüning and Schubert, 2022). These ads, while evoking some compassion, focused mainly on the common goals, common destiny, common identity, and unity of the party and the nation. This is thus an additional path through which climate change ads can increase kama muta—by focusing on unity, identity, and belonging in the climate movement through common goals and common destiny (Stollberg and Jonas, 2021). Indeed, collective action for forest protection and against climate change is predicted by being moved about collective efficacy (Landmann and Rohmann, 2020). We, therefore, expect that climate-related media content can evoke kama muta, which in turn increases motivation to mitigate climate change.

### Overview of studies

We conducted four preregistered studies to test the relationship between feelings of kama muta and pro-environmental attitudes, intentions, and behavior. An overview of procedures and measures across these four studies is provided in Table 1. To provide convergent validity, we used different videos (Studies 1 and 4) and audio clips (Studies 2 and 3) that focused on climate change-related aspects. Next to assessing pro-environmental intentions across all studies, we measured actual behavior as operationalized by time spent on reading about climate-related information (Studies 1, 2, and 4), visiting external websites related to climate change (Study 1), or donating money (Study 4). Across all studies, we tested similar registered hypotheses focusing on the relationship between feelings of kama muta and pro-environmental intentions and behavior, as well as on the effects of controlling for attitudes. As hypotheses depended on different designs, we report the respective hypotheses at the beginning of each study.

**Table 1:** Overview of the stimuli and measures used in Studies 1–4.

	Participants	Design	Stimuli	Main Predictor		Outcome		Attitudes	Additional Items
				Kama Muta	Other Emotions	Intentions	Behavior		
Study 1	Norwegian undergraduates, US Amazon MTurk, *N* = 224 (99 females, 3 other/missing), 17–69 (*M* = 31.84, SD = 10.55)	Correlational	Video clip (randomly chosen from two videos)	KAMMUS-S (Zickfeld et al., 2019) Sensations (3 items; *moist eyes/cried, goosebumps/chills, warm feeling in the center of the chest*) Labels (3 items; *moved*, *touched*, *heartwarming*) → Kama Muta Index CS Appraisal (4 items; *incredible bond, exceptional sense of closeness, unique kind of love, welcoming or being welcomed*)	*Angry*, *anxious* (1 item each)	Pro-environmental intentions (11 items) (Dietrich, 2013)	Reading time (s); links clicked	INS Scale (Schultz, 2001); New Ecological Paradigm (NEP; 6 items; (Dunlap et al., 2000, p. 200)); Climate change attitude survey (CCAS; 17 items; only US sample; (Christensen and Knezek, 2015)); General Climate Attitude (2 items)	
Study 2	US Amazon MTurk; *N* = 220 (97 females, 2 other/missing); 19–70 (*M* = 35.14, SD = 10.03)	Between subjects (personal story vs. neutral story)	Audio clip		*Anger* (3 items; *angry, outraged, furious)*, *Fear* (3 items; *fearful, anxious, frightened*)*, Sadness* (3 items; *sad, dejection, depressed), Awe* (1 item)	Climate Intention Scale Learn more (6 items) Discuss and share (8 items) Change behavior (6 items) Support climate policies/groups (6 items)	Reading time (s)	CCAS	Interpersonal Reactivity Index-EC IRI-EC; 7 items (Davis, 1983), Prior Mood (6 items)
Study 3	Norwegian undergraduates; *N* = 220 (166 females, 2 other/missing); 18–57 (*M* = 23.37, SD = 5.70)	Between subjects (personal story vs. neutral story)	Audio clip		*Anger*, *Fear, Sadness,*	Climate Intention Scale (10 items) General intentions (7 items) Sharing with others (3 items)		CCAS (shortened 9 items)	Attitudes toward immigrants (5 items); Attitudes toward climate refugees
Study 4	US Prolific.ac; *N* = 385 (181 females, 2 other); 18–73 (*M* = 36.27, SD = 11.98)	Between subjects (emotional vs. Neutral climate change video)	Video clip		*Anger*, *Fear, Sadness, Hope* (3 items; *hopeful, optimistic, encouraged*), *Feeling Manipulated* (1 item)		Reading time (s); Donation ($0 to $1)	CCAS (17 items)	IRI-EC

#### Transparency and openness

All studies were conducted between 2017 and 2019. We report how we determined our sample size, all data exclusions (if any), all manipulations, and all measures in the study. All data, syntax, materials, and preregistrations can be accessed at https://osf.io/fsb4n/. There, we also specify the packages and versions used, which is also detailed in the Supplemental material. In addition, a detailed overview of all items and questionnaires can be found on the project page. All studies were preregistered (https://osf.io/e6dgq/ and https://osf.io/ypr7e) and we explicitly state deviations from the preregistration and distinguish between confirmatory and exploratory analyses. For all statistical analyses, the alpha level was set at 0.05. All studies were evaluated and approved by the ethical review board of the University of Oslo.

## Study 1

Study 1 was designed to test two pre-registered hypotheses: **H1**. Kama muta evoked by a pro-environmental message predicts intentions of pro-environmental behavior and actual behavior positively. The more reported kama muta, the more intention and actual behavior. **H2**. The association of kama muta with intentions of pro-environmental behavior and actual behavior is moderated by environmental attitude. The more pro-environmental a person’s attitude is before seeing the message, the more her experience of kama muta will increase intention and actual behavior.

### Method

#### Participants

Power analyses for an expected small to medium effect (*r* = 0.20)^1^ resulted in 150 participants with a power of 0.80 and an alpha level of 0.05 using G^*^Power 3 (Faul et al., 2007). Based on recent recommendations for correlational studies, we aimed to sample at least 160 participants (Schönbrodt and Perugini, 2013).

A total of 142 psychology undergraduates were recruited from the University of Oslo in exchange for partial course credit. They completed the study in Norwegian. Based on pre-registered exclusion criteria, 62 participants were excluded from this study if they did not attempt at least 50% of the questionnaire or/and did not watch the whole video clip. In addition, 163 participants were recruited from Amazon Mturk, offering compensation of $1.60. Based on the same exclusion criteria, 19 participants were excluded from this study. These participants completed the study in English. The final sample consisted of 224 participants (99 females, 1 non-binary, and 2 missing), aged 17 to 69 years (*M* = 31.84, SD = 10.55).

#### Design

The research design of the study was correlational as it examined the relationship between (1) kama muta and intentions of pro-environmental behavior and (2) kama muta and actual behavior. After providing informed consent, participants were shown one video clip (about 2 min each) about environmental concerns selected randomly from two video clips preselected by the research team to be moving (see Supplementary material for video clips). One video clip featured the spoken word artist and activist Prince Ea recounting how humans have damaged the planet and emphasizing that change can only be achieved if all stand together, highlighting connectedness. The second video clip, narrated by Morgan Freeman, told the story about a sustainable future planet, highlighting that it can only be achieved if humans work together and start acting. Participants were then asked to complete a questionnaire after watching the video clip.

#### Measures

##### Climate attitudes

Before the video clip, participants completed the pictorial *EINS* scale, which included seven circles increasing in overlap (Schultz, 2001). Participants were instructed to choose the circle formation that best described their relationship with the natural environment. Afterward, we assessed environmental values using a shortened version of the New Ecological Paradigm (NEP) scale (Whitmarsh, 2011) including six items (e.g., “Humans have the right to modify the natural environment to suit their needs”) on a scale ranging from 1 (*Strongly disagree*) to 5 (*Strongly agree*) (*α* = 0.79). A short measure of general climate attitude was based on two items: “How concerned are you about climate change?” and “In general, should [Norway/the US] as a country protect the climate at the expense of loss of income (e.g., oil),” which were completed on two 7-point scales from 1 (*Not at all*) to 7 (*Very much*) and 1 (*Strongly disagree*) to 7 (*Strongly agree*) (*α* = 0.87). US participants also completed the 15 items of the Climate Change Attitude Survey (CCAS) (Christensen and Knezek, 2015) (e.g., “I am concerned about global climate change.”; *α* = 0.93) on a 5-point scale.

##### Kama Muta and other emotions

After watching the video clip, participants’ feelings about kama muta were assessed using a short form of the Kama Muta Multiplex Scale (KAMMUS-S; Zickfeld et al., 2019). We included three items on sensations (“moist eyes or cried,” “chills or goosebumps,” and “warm feeling in the chest”), four items targeting appraisals (e.g., “I felt or observed an incredible bond”), and three items measuring emotion labels (“It was heartwarming,” “I was moved,” and “I was touched”). In addition, we included two items asking whether the video made participants feel angry or anxious. All responses were made on 7-point scales ranging from 0 (*Not at all*) to 6 (*A lot*). Items targeting sensations and emotion labels were averaged into a kama muta score (*α* = 0.91) and items targeting appraisals into a communal sharing score (*α* = 0.94).

##### Pro-Environmental intentions and behavior

Afterward, participants answered 11 items on their environmental intentions (“Based on your environmental concern, how likely are you to,” e.g., “reduce your meat consumption,” “become active in an environmental organization”; *α* = 0.92) on Likert scales from 1 (*Very unlikely*) to 5 (*Very likely*) (adapted from Dietrich, 2013). These items were averaged into an intention index. Finally, participants were presented with country-specific information on climate change including three links to (1) information sites, (2) petition links, and (3) donation websites. The actual behavior of the participants was measured based on the number of links clicked in the study about (1) information sites, (2) petitions links, and (3) donation websites. In addition, reading time spent on the page presenting the links was calculated and demographic information was collected.

### Results

As pre-registered, reading time spent on the information page was transformed using a log transformation. Regarding clicking behavior, the majority of the US sample (more than 95%) did not click on any link(s), and the clicking behavior was not recorded in the Norwegian sample due to technical issues, which did not allow us to perform any models on this variable.^2^ For all analyses, we controlled for the type of sample by including the country (0, US; 1: Norway) and its interaction with the main predictors in the model.^3^ An overview of correlations among the main variables is presented in Supplementary Tables S1, S2.

#### Confirmatory (registered) results

**Figure 1:**
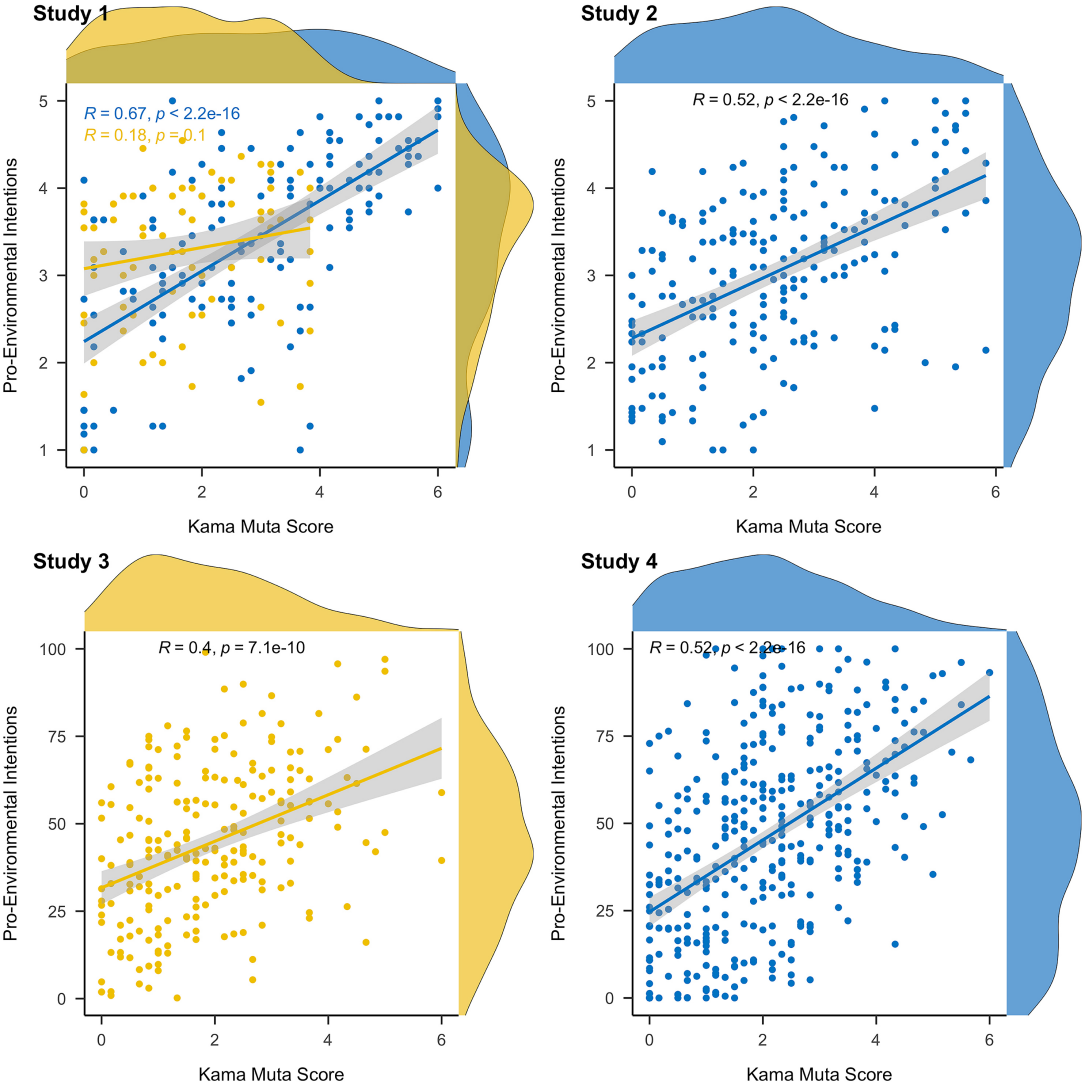
Overview of the relationship between kama muta and pro-environmental intentions across the four studies. Gray shaded area indicates 95% confidence intervals. Blue represents the US and yellow is Norway.

For H1, we performed linear regression with pro-environmental intention as the dependent variable, the kama muta score as the independent variable, and the country as a factor and its interaction with the kama muta score (Table 2). We found that experiencing kama muta positively predicted pro-environmental intentions, *b* = 0.45, *t*(222) = 6.31, *p <* 0.001. The country also positively predicted pro-environmental intentions with Norwegians’ ratings being on average higher than the US-Americans, *b* = 0.41, *t*(222) = 4.12, *p <* 0.001. Finally, we found an interaction effect between the kama muta score and country, *b* = −0.48, *t*(222) = −3.39, *p <* 0.001. The effect was stronger for the US-Americans (*r* = 0.67 [0.57, 0.76]) as compared to the Norwegians (*r* = 0.18 [−0.03, 0.40]) (Figure 1).

**Table 2:** Regression results using intention and reading time as the criterion in Study 1.

Predictor	*b*	*b* 95% CI	*sr* ^2^	*sr*^2^ 95% CI	Fit
*Pro-Environmental Intentions*					
(Intercept)	2.24^**^	[1.99, 2.49]			
Kama Muta	0.40^**^	[0.33, 0.48]	0.34	[0.24, 0.44]	
Country	0.83^**^	[0.44, 1.23]	0.05	[0.00, 0.10]	
Kama Muta ^*^ Country	−0.28^**^	[−0.45, −0.12]	0.03	[−0.00, 0.07]	
					*R*^2^ = 0.348^**^
					95% CI [0.24,0.43]
*Reading Time (log)*					
(Intercept)	2.03^**^	[1.71, 2.35]			
Kama Muta	0.10	[−0.00, 0.19]	0.01	[−0.01, 0.04]	
Country	1.32^**^	[0.79, 1.85]	0.08	[0.02, 0.14]	
Kama Muta ^*^ Country	0.05	[−0.17, 0.27]	0.00	[−0.01, 0.01]	
					*R*^2^ = 0.291^**^
					95% CI [0.19,0.37]

A significant *b*-weight indicates the semi-partial correlation is also significant. *b* represents unstandardized regression weights. *sr*^2^ represents the semi-partial correlation squared. The lower and upper limits of the confidence intervals are indicated in brackets. ^*^Indicates *p* < 0.05. ^**^Indicates *p* < 0.01.

In addition, we performed linear regression with reading time as the dependent variable, the kama muta score as the independent variable, and the country as a factor and its interaction with the kama muta score (Table 2). We found that experiencing kama muta positively predicted actual reading time, *b* = 0.16, *t*(222) = 2.20, *p =* 0.029. The country also positively predicted reading time with Norwegian times being on average higher than the ones of the US sample, *b* = 0.53, *t*(222) = 4.92, *p <* 0.001. Finally, we observed no significant interaction effect between the kama muta score and the country, *b* = 0.02, *t*(222) = 0.47, *p =* 0.640 (US: *r =* 0.16 [0.00, 0.32]; NO: *r =* 0.16 [−0.05, 0.38]).

For H2, we performed the same linear regression with pro-environmental intention as the dependent variable and the kama muta score as the independent variable. We added climate attitude as an additional predictor and its interaction with the kama muta score to the model. In addition, we also controlled for the country as in the previous models, which can be found in the (Supplementary Table S4). For climate attitude, we used four different measures in four separate models: (1) general climate attitude, (2) NEP, (3) EINS, and (4) CCAS (US only). A detailed overview can be found in the (Supplementary Table S4). When focusing on pro-environmental intentions as the dependent variable, we observed that all attitude measures showed significant positive effects. In addition, kama muta predicted pro-environmental intentions positively when controlling for climate attitudes across all four models. We observed a statistically significant interaction effect for the NEP and EINS attitude measures, but not for the general climate attitude index or the CCAS. The observed interaction effects did not support our predictions. Instead of climate attitude moderating the strength of kama muta on pro-environmental intentions, kama muta moderated the strength of climate attitudes on intentions: Attitudes had a stronger effect on intentions for low levels of kama muta than for high levels. Note that this effect was small and not consistently found across all measures though. For reading time as the outcome variable, we observed statistically significant positive effects by climate attitude for all measures except the CCAS. Kama muta showed positive predictions across all models except CCAS, but was not statistically significant in any of these. We did not observe any statistically significant interaction effects (see Supplementary Figures S3, S4).

#### Exploratory results regarding other emotions

We explored the influence of other emotions, by repeating a model with pro-environmental intentions as the outcome and climate attitudes, and the kama muta score and the angry and anxious items as predictors. Across all models (four different climate attitude measures), the kama muta score positively predicted pro-environmental intentions when controlling for prior climate attitudes and other emotions. Feeling angry or anxious did not show any significant predictions across the models, except for angry which significantly positively predicted intentions when controlling for the EINS measure (see Supplementary Table S5).

### Discussion

The first study presented a correlational test of the hypothesis that feelings of kama muta evoked by videos on climate change are associated with pro-environmental intentions. We found that kama muta was indeed positively associated to act pro-environmentally, also when controlling for prior pro-environmental attitudes. This effect was smaller in the Norwegian sample, which might be because the Norwegian participants showed lower kama muta ratings for the videos than the US sample. We did not find evidence that this effect was higher for participants endorsing strong attitudes on the importance of addressing climate change. Instead, we observed that strong experiences of kama muta were associated with higher pro-environmental intentions regardless of participants’ prior attitudes. Although this finding was not consistent across climate attitude measures, we will focus on replicating the pattern in Study 2 to say more about its validity. In addition, we also found a small positive association with how long participants spent on a page presenting pro-environmental information used as a proxy for actual pro-environmental behavior.

Unfortunately, another behavior measure we tried, namely whether participants clicked on links, was not usable due to a technical failure in the Norwegian sample. In the US sample, there was a low percentage of clicking these links. It seems that other aspects, such as time constraints in finishing the study on Amazon MTurk, had a stronger importance than experiences of kama muta.

While we presented a first correlational test of our main hypothesis in Study 1, ads about climate change are likely to evoke a spectrum of different emotions. Thus, a possibility is that any strong emotion, such as anger or anxiety, could increase pro-environmental intentions. Our exploratory correlational results in the present study did not support this idea. However, in Study 2, we controlled for other emotional reactions in a more systematic fashion. In addition, we wanted to test an experimental effect of kama muta on pro-environmental intentions moving beyond the correlational findings of the first study.

## Study 2

Does listening to a personal account of a victim of climate change-related floods lead to more intention for various types of climate action than a more impersonal account of the same event independent of prior climate attitude and other emotions evoked? Is this effect mediated by increased kama muta? To answer these questions, we ran Study 2 with the following pre-registered hypotheses: **H1**. The type of story (personal vs. neutral) influences the amount of kama muta experienced such that participants feel the most kama muta in the personal condition; **H2**. The amount of kama muta evoked by the story predicts (a) intention and (b) reading time when controlling for prior climate attitude, evoked anger, sadness, and anxiety; **H3**. When controlling for the type of story (personal vs. neutral), kama muta significantly predicts intentions and reading time.

### Method

#### Participants

Before data collection, we performed a power analysis for a mediation model expecting small effects (*β* = 0.20) setting the power at 0.80 and the alpha level at 0.05. Employing an application by Schoemann et al. (2017), we obtained a final sample size recommendation of 255 participants.

Two hundred and fifty-eight participants were recruited online from Amazon MTurk. Based on exclusion criteria, 26 participants were excluded from this study as they did not attempt at least 70% of the questionnaire (*n =* 1) or/and did not listen to the whole audio clip, which was recorded with a timer (*n =* 26). In addition, 16 participants indicated that they experienced technical difficulties with audio playback. Although not registered, we also excluded these participants as many of them indicated that they could not hear the full audio clip.^4^ The final sample size consisted of 220 participants (97 females and 2 non-binary), aged 19 to 70 years (*M* = 35.14, SD = 10.03). Participants received $2.40. They completed the study in English.

#### Design

In contrast to Study 1, we employed a between-subjects design with two different audio clips (*personal story, n =* 112, or *neutral story, n =* 108). In both conditions, participants listened to a short story about a typhoon in the Philippines. In the personal story condition, participants heard an account of the flood with a story of the personal connection between mother and daughter and of finding a new family in the global climate change movement. In the neutral story condition, participants heard a summary of the flood and a brief extract from a speech of the Philippine representative at the FN summit in New York.

#### Materials

##### Climate attitudes

Before the audio clip, participants indicated their concern about the climate using the CCAS as used in Study 1 (*α* = 0.96).

##### Prior mood

The mood of the participants was also evaluated based on six items (“I’m stressed,” “I worry,” “I’m alert,” “I have difficulty focusing,” “I feel good,” and “I feel bad”) on a 5-point scale from 1 (not at all) to 5 (completely).

##### Kama muta and other emotions

After hearing the audio clip, participants completed the same kama muta items with regard to sensations, appraisal, and labels as in Study 1, again computing the same kama muta (*α* = 0.89) and communal sharing scores (*α* = 0.89). Anger (*α* = 0.93), fear (*α* = 0.90), and sadness (*α* = 0.77) responses were measured with three items each. We also included an item measuring awe. All responses were completed on the same 7-point scale ranging from 0 (Not at all) to 6 (Very much). Afterward, participants completed questions regarding the topic of the audio clip and whether they had experienced technical problems.

##### Intentions

Participants then indicated their climate-action intentions in response to 26 items created for the current study and based on previous intention scales such as the one from Study 1. The idea was to cover a range of different climate-action intentions, personal, political, practical, and communicative, focused on climate change mitigation specifically. An exploratory factor analysis indicated a four-factor solution (see Supplementary Figure S1). Five items were dropped because they loaded on several factors and the final four factors focused on intentions (1) to learn more about climate change (*α* = 0.96), consisting of six items, e.g., “I am interested in seeking out information about how the environment is impacted by humans”; (2) to discuss the report and share it with others (*α* = 0.95; six items), e.g., “I intend to discuss the clip with others”; (3) to change one’s personal behavior (*α* = 0.86; four items), e.g., “I intend to eat less meat”; and (4) to support climate policies or groups (*α* = 0.91; five items), e.g., “I would volunteer or campaign for an organization aiming to reduce global warming.” The items were answered on a Likert scale from 1 (*not at all true*) to 5 (*completely true*) or 1 (*strongly disagree*) to 5 (*strongly agree*). Instructions asked participants to “indicate to what extent the following statements are true of you right now.” An intention index was calculated by averaging all items (*α* = 0.97).

##### Trait Empathic Concern

Afterward, participants completed the seven-item trait empathic concern subscale of the Interpersonal Reactivity Index (IRI; Davis, 1983) on a Likert scale from 1 (*Does not describe me well*) to 5 (*Describes me very well*) (*α* = 0.94).

##### Reading behavior

After completing the demographic information, participants were provided with information related to the audio clip and climate change in general. They were instructed that they could finish the study or read through the information. We recorded how long participants stayed on the page presenting the information using a timer.

### Results

Again, as pre-registered the reading time variable was log-transformed before analysis. As in Study 1, we employed two measures assessing intentions and behavior: (1) the intention index (2) and the reading time measure. An overview of correlations among the main variables is provided in Supplementary Table S6.

#### Confirmatory results

For H1, we performed an independent samples Welch’s t-test with the kama muta score as the dependent variable and condition as the independent variable. We found that the kama muta score differed significantly for condition, Cohen’s *d* = 0.52 [0.25, 0.80], *t*(218) = 3.90, *p <* 0.001. On average, the personal story (*M* = 2.77, SD = 1.55) evoked higher scores than the neutral story (*M* = 1.98, SD = 1.49). In an exploratory fashion, we found a similar effect for communal sharing appraisal ratings (*d =* 0.70 [0.43, 0.98], *t*(213.26) = 5.21, *p* < 0.001, *M_neutral =_* 1.52, SD *=* 1.72, *M_km =_* 2.67, SD *=* 1.53).

For H2, we performed linear regression with intention as the dependent variable and prior climate attitudes, kama muta score, anger, sadness, and anxiety indices as the independent variables. An overview of the results is presented in Table 3. When controlling for prior climate attitude, anger, sadness, and anxiety, we found that experiencing kama muta positively predicted intentions (see Figure 1). Second, we found that anger predicted intentions positively and sadness negatively. Third, we found that climate attitudes also positively predicted intentions. On the other hand, anxiety did not predict intentions significantly.

**Table 3:** Regression results using intentions as the criterion in Studies 2–4.

Predictor	*b*	*b* 95% CI	*β*	*β* 95% CI	*sr* ^2^	*sr*^2^ 95% CI	*r*	Fit
*Study 2*								
(Intercept)	0.09	[−0.33, 0.52]						
Climate Attitude	0.59^**^	[0.49, 0.70]	0.51	[0.42, 0.60]	0.25	[0.17, 0.33]	0.57^**^	
Kama Muta	0.19^**^	[0.13, 0.28]	0.33	[0.21, 0.45]	0.06	[0.02, 0.10]	0.52^**^	
Anger	0.16^**^	[0.11, 0.25]	0.30	[0.18, 0.42]	0.05	[0.01, 0.09]	0.48^**^	
Sadness	−0.11^*^	[−0.19, −0.03]	−0.17	[−0.31, −0.04]	0.01	[−0.01, 0.03]	0.39^**^	
Anxiety	0.04	[−0.02, 0.13]	0.11	[−0.03, 0.24]	0.00	[−0.01, 0.02]	0.46^**^	
								*R*^2^ = 0.59^**^
								95% CI [0.50,0.64]
*Study 3*								
(Intercept)	−28.00**	[−42.15, −9.88]						
Climate Attitude	0.75^**^	[0.58, 0.92]	0.48	[0.37, 0.58]	0.22	[0.13, 0.30]	0.54^**^	
Kama Muta	5.21^**^	[2.96, 7.45]	0.31	[0.18, 0.45]	0.06	[0.01, 0.11]	0.40^**^	
Anger	2.66	[−0.47, 5.80]	0.13	[−0.02, 0.29]	0.01	[−0.01, 0.03]	0.28^**^	
Sadness	−1.04	[−3.92, 1.84]	−0.07	[−0.27, 0.13]	0.00	[−0.01, 0.01]	0.25^**^	
Anxiety	−0.63	[−3.45, 2.19]	−0.04	[−0.22, 0.14]	0.00	[−0.00, 0.01]	0.23^**^	
								*R*^2^ = 0.40^**^
								95% CI [0.23,0.46]
*Study 4*								
(Intercept)	−37.99**	[−50.18, −25.79]						
Climate Attitude	12.78**	[9.62, 15.93]	0.32	[0.24, 0.40]	0.09	[0.04, 0.13]	0.49^**^	
Kama Muta	5.06**	[2.87, 7.24]	0.26	[0.15, 0.37]	0.03	[0.00, 0.05]	0.52^**^	
Anger	2.06*	[0.26, 3.86]	0.12	[0.02, 0.23]	0.01	[−0.00, 0.02]	0.36^**^	
Sadness	1.05	[−1.21, 3.31]	0.06	[−0.07, 0.19]	0.00	[−0.00, 0.01]	0.39^**^	
Anxiety	2.81*	[0.71, 4.92]	0.17	[0.04, 0.29]	0.01	[−0.00, 0.02]	0.48^**^	
Hope	2.81**	[1.01, 4.61]	0.17	[0.06, 0.28]	0.01	[−0.00, 0.03]	0.43^**^	
								*R*^2^ = 0.51^**^
								95% CI [0.44,0.56]

A significant *b*-weight indicates the *β*-weight and semi-partial correlation are also significant. *b* represents unstandardized regression weights. *β* indicates the standardized regression weights. *sr*^2^ represents the semi-partial correlation squared. *r* represents the zero-order correlation. The lower and upper limits of the confidence intervals are indicated in brackets. ^*^Indicates *p* < 0.05. ^**^Indicates *p* < 0.01.

We then performed the same analysis with reading time as the dependent variable (see Table 4). When controlling for climate attitude, anger, sadness, and anxiety, we found that experiencing kama muta did not significantly predict reading time, nor did anger or anxiety. However, we observed that sadness positively predicted reading time, and so did climate attitude. An exploratory model with only the kama muta score as the predictor indicated that kama muta positively predicted reading time with a similar effect as observed in Study 1, *b* = 0.15, *t*(218) = 2.21, *p =* 0.028.

**Table 4:** Regression results using reading time (log) as the criterion in Study 2.

Predictor	*b*	*b* 95% CI	*β*	*β* 95% CI	*sr* ^2^	*sr*^2^ 95% CI	*r*	Fit
(Intercept)	3.09^**^	[2.48, 3.71]						
Climate Attitude	0.19^*^	[0.04, 0.34]	0.17	[0.04, 0.30]	0.03	[−0.01, 0.07]	0.21^**^	
Kama Muta	0.02	[−0.08, 0.13]	0.04	[−0.14, 0.22]	0.00	[−0.01, 0.01]	0.15^*^	
Anger	0.03	[−0.07, 0.13]	0.05	[−0.12, 0.22]	0.00	[−0.01, 0.01]	0.12	
Sadness	0.15^*^	[0.03, 0.27]	0.25	[0.05, 0.44]	0.03	[−0.01, 0.07]	0.22^**^	
Anxiety	−0.09	[−0.20, 0.02]	−0.16	[−0.37, 0.04]	0.01	[−0.02, 0.04]	0.09	
								*R*^2^ = 0.09^**^
								95% CI [0.02,0.15]

A significant *b*-weight indicates the *β*-weight and semi-partial correlation are also significant. *b* represents unstandardized regression weights. *β* indicates the standardized regression weights. *sr*^2^ represents the semi-partial correlation squared. *r* represents the zero-order correlation. The lower and upper limits of the confidence intervals are indicated in brackets. ^*^Indicates *p* < 0.05. ^**^Indicates *p* < 0.01.

For H3, we performed linear regression with intention as the dependent variable, climate attitude, kama muta, anger, sadness, and anxiety as independent variables, and condition as a factor. We found that experiencing kama muta positively predicted intentions, *b* = 0.38, *t*(213) = 6.10, *p <* 0.001, and so did anger, *b* = 0.28, *t*(213) = 4.77, *p <* 0.001, and prior climate attitudes, *b* = 0.51, *t*(213) = 11.59, *p <* 0.001. Sadness predicted intentions negatively, *b* = −0.16, *t*(213) = −2.45, *p =* 0.015. In addition, the personal story became a negative predictor of intentions when controlling for experienced kama muta and other variables, *b* = −0.13, *t*(213) = −2.90, *p =* 0.004. Again, anxiety, *b* = 0.08, *t*(213) = 1.19, *p =* 0.236, did not predict intentions significantly.

Using condition as the sole predictor, we found only a small non-significant effect of our manipulation in the opposite direction for both intentions, *d =* −0.07 [−0.33, 0.20], *t*(215.67) = −0.49, *p =* 0.62, and reading time, *d =* −0.06 [−0.32, 0.21], *t*(217.86) = −0.43, *p =* 0.67, suggesting slightly more intentions and reading time after the neutral story. In a mediational analysis, however, we observed that the personal story (vs. the neutral story) had a positive indirect effect on intentions *via* experiencing kama muta, *b* = 0.14, *B* = 0.28 [95% Bootstrap CI, 0.14, 0.44]. This indirect effect was held when controlling for prior climate attitudes, anxiety, sadness, and anger.

In addition, we performed linear regression with reading time as the dependent variable, climate attitudes, kama muta, anger, sadness, and anxiety as the independent variables, and condition as a factor. When controlling for condition, climate attitude, anger, sadness, and anxiety, we found that experiencing kama muta did not significantly predict reading time, *b* = 0.07, *t*(213) = 0.78, *p =* 0.435. Second, neither anger, *b* = 0.04, *t*(213) = 0.40, *p =* 0.691, nor anxiety, *b* = −0.18, *t*(213) = −1.72, *p =* 0.086, nor condition, *b* = −0.08, *t*(213) = −1.20, *p =* 0.232, predicted reading time significantly. However, we observed that sadness positively and statistically significantly predicted reading time, *b* = 0.25, *t*(213) = 2.55, *p =* 0.012, which also held for climate attitudes, *b* = 0.17, *t*(213) = 2.54, *p =* 0.012.

Similar to the previous model, we observed that kama muta mediated the effect of condition on reading time, *b* = 0.04*, B* = 0.08 [95% Bootstrap CI: 0.02, 0.19] (Supplementary Table S15). However, this indirect effect did not hold when controlling for climate attitude or evoked sadness.

#### Exploratory results

We repeated a regression analysis to explore whether climate attitudes moderated the association between kama muta and intentions. In contrast to Study 1, we found no significant interaction effects for the intention score or for reading time. We observed a small non-significant interaction effect for reading time suggesting that the effect of kama muta on reading time was stronger for participants high on pro-environmental attitudes, failing to replicate patterns from Study 1 (see Supplementary Figures S3, S4). Similarly, we tested a possible moderation by trait empathic concern but did not find significant interaction effects (see Supplementary Table S8).

We ran another mediation model with the story (personal vs. neutral) as the predictor, intentions as the outcome, and communal sharing as the mediator. We observed that communal sharing partially mediated the effect, *b* = 0.13, *B =* 0.26 [95% Bootstrap CI: 0.15, 0.40], while the negative effect of condition on intentions again became stronger (Supplementary Table S15).

Finally, we explored the association of different intention subscales with kama muta. We observed that kama muta ratings showed stronger relationships to share information (*r =* 0.58 [0.48, 0.66]) than to seek information related to global warming (*r* = 0.44 [0.33, 0.54]), to act by reducing one’s carbon footprint (*r =* 0.35 [0.22, 0.46]) or supporting environmental organizations (*r =* 0.32 [0.20, 0.43]).

### Discussion

In Study 2, we replicated our main findings from Study 1 and expanded our findings by providing an experimental test of our hypothesis and controlling more systematically for other experienced emotions. Again, we found that kama muta was associated with an increase in climate-action intentions and reading time. The association with intentions also held when controlling for prior attitudes toward climate change, as well as feelings of anger, anxiety, and sadness. However, this was not the case for the association of kama muta with reading time. Regarding a potential moderation of these effects by prior climate attitudes, we found different patterns in Studies 1 and 2. In Study 3, we will explore a potential moderation again.

We did not find evidence that our experimental manipulation of a personal versus a neutral story about a typhoon influenced intentions or reading time. However, we found evidence that this was the case indirectly *via* feelings of kama muta (and increased communal sharing). In Study 3, we aimed at replicating these findings using different measures and a more neutral control condition to provide convergent validity of our findings.

## Study 3

Does listening to a personal account of a victim of climate change-related floods lead to more intention for various types of climate action than a control audio file independent of prior climate attitude and other emotions evoked? Is this effect mediated by increased kama muta? We tested the same hypotheses as in Study 2: **H1**. The type of story (personal vs. neutral) influences the amount of kama muta experienced such that participants feel the most kama muta in the personal condition. **H2**. The amount of kama muta evoked by the story predicts intention when controlling for prior climate attitude, evoked anger, sadness, and anxiety. **H3**. When controlling for the type of story (personal vs. neutral), kama muta significantly predicts intentions.

### Method

#### Participants

Based on the findings from the previous study, we performed an *a priori* power analysis for a mediation model using an online application expecting small effects (path a: *b* = 0.20, path b: *b* = 0.35) setting the power at 0.80 and the alpha level at 0.05. We obtained a final sample size recommendation of 185 participants.

A total of 370 undergraduate participants were recruited through a subject pool at the University of Oslo. Based on pre-registered exclusion criteria, 150 participants were excluded from this study as they did not attempt at least 70% of the questionnaire (*n* = 14), did not listen to the whole audio clip which was recorded with a timer (*n* = 115), and/or indicated that they do not want their answers to be used for the final analysis (and only participate for educational purposes, *n* = 44).^5^ The final sample size consisted of 220 participants (166 females, 52 males, 1 other, and 1 missing), aged 18 to 57 years (*M* = 23.37, SD = 5.70). The majority indicated Norwegian as their nationality (190). Participants received partial course credit for participating in the study. The majority completed the study in Norwegian, while eight participants chose the English version.

#### Design

The research design of this study was again a between-subjects design. We again employed an audio clip that either presented a similar personal story about a typhoon in the Philippines as in Study 2 or a neutral story of a TED talk focusing on trying something new each day for a month (*personal story, n =* 100, or *neutral story, n =* 120).

#### Measures

##### Climate attitudes

Before the audio clip, participants indicated their concern about the climate on a short version of the CCAS with nine items (e.g., “I am concerned about global climate change.”), with slider scales (*α* = 0.80) ranging from 1 (Strongly disagree) to 100 (Strongly agree).^6^ We also assessed the participants’ attitudes toward refugees in Norway with five items (*α* = 0.76), e.g., *“Most immigrants make an important contribution to Norwegian working life*.*”* (adapted from Blom, 2017).

##### Kama muta and other emotions

After the audio clip, participants completed the same measures with regard to kama muta labels, physiology, communal sharing appraisals, anger, sadness, and anxiety, as in Study 2. We calculated the same kama muta (*α* = 0.85), communal sharing (*α* = 0.85), anger (*α* = 0.90), sadness (*α* = 0.85), and anxiety (*α* = 0.92) indices as in Study 2.

##### Pro-Environmental Intentions

We then included 10 items to assess intentions to act upon climate change (*α* = 0.88; “After hearing the story, to what extent do you intend to:” e.g., “Volunteer or campaign for an organization aiming to reduce global warming”) using a slider scale format ranging from 0 (*Very unlikely*) to 100 (*Very likely*). This was a short form of the 26-item version from Study 2, which we formed by keeping items from each sub-scale that showed good item-total correlations. We performed a factor analysis to determine different aspects of climate intentions. A parallel analysis suggested a two-factor solution. Seven items loaded on the first factor presented climate intentions in general (*α* = 0.87), while three items loaded on the second factor presented intentions to share the message with other individuals (*α* = 0.87; see Supplementary Figure S2).

Finally, participants completed several items on attitudes toward (climate) refugees not focal to the current purposes.^7^ In the end, participants completed demographic information and were debriefed.

### Results

We repeated the same pre-registered analyses as in Study 2, except those referring to reading time. We also performed analyses separately on this index for the general intentions and the intentions to share the message with others, which can be found in Supplementary Table S10. An overview of correlations among the main variable is provided in Supplementary Table S9.

#### Confirmatory results

For H1, we performed a Welch’s t-test with the kama muta score as the dependent variable and condition as the independent variable. On average, the personal story (*M* = 2.60, SD = 1.30) evoked higher kama muta scores than the neutral story (*M* = 1.35, SD = 1.03), Cohen’s *d =* 1.08 [0.79, 1.36], *t*(186.92) = 7.78, *p* < 0.001. Similarly, the personal story (*M =* 2.72, SD *=* 1.40) evoked higher communal sharing ratings than the neutral story (*M =* 1.07, SD *=* 1.23), *d =* 1.27 [0.97, 1.56], *t*(198.88) = 9.25, *p* < 0.001.

For H2, we performed linear regression with intention as the dependent variable and climate attitudes, kama muta score, anger, sadness, and anxiety indices as the independent variables as in Study 2. An overview of the results is presented in Table 3. When controlling for climate attitude, anger, sadness, and anxiety, we found that experiencing kama muta positively predicted intentions (see also Figure 1). In addition, climate attitudes also positively predicted intentions. On the other hand, anger, sadness, and anxiety did not predict intentions significantly.

We repeated the same model and added condition as an additional predictor. Again, intentions were positively predicted by climate attitudes (*b* = 0.46 [0.35, 0.57]), kama muta (*b* = 0.32 [0.19, 0.46]), and this time also anger (*b* = 0.16 [0.01, 0.32]). Sadness (*b* = 0.07 [−0.15, 0.29]) and fear (*b* = −0.01 [−0.19, 0.17]) did not show statistically significant effects. Finally, condition predicted intentions negatively (*b* = −0.25 [−0.41, −0.10]).

As in Study 2, we did not observe a statistical significant effect of our manipulation on intentions, *t*(209.37) = 1.06, *p =* 0.291. The personal story evoked higher intentions (*M =* 46.19, SD = 22.0) than the neutral story (*M =* 43.05, SD *=* 21.6), though this effect was rather small and statistically non-significant, *d =* 0.14 [−0.12, 0.41].

#### Exploratory results

Again, we observed that experiencing kama muta mediated the effect of personal vs. neutral conditions on intentions with an indirect effect of *b* = 0.22, *B* = 9.72 [95% Bootstrap CI: 6.54, 14.22] (Supplementary Table S15). This indirect effect held true when controlling for prior climate attitudes, anxiety, sadness, and anger. Kama muta ratings showed a slightly stronger correlation with general pro-environmental intentions (*r* = 0.37 [0.25, 0.48]) compared to sharing information with others (*r* = 0.34 [0.22, 0.45]). Finally, we did not observe a significant moderation effect of climate attitudes on the relationship between kama muta and pro-environmental intentions (Supplementary Figure S3).

### Discussion

We replicated our findings from Study 2 that kama muta positively predicts climate-action intentions when controlling for other emotions and climate attitudes. Again, we found only a small and non-significant direct effect of the experimental condition on intentions and observed that this relationship was mediated by felt kama muta as in Studies 1 and 2. In Study 4, we employed two types of video stimuli on climate change to study their direct and indirect effects on intentions *via* kama muta. So far, we have also mainly focused on studying pro-environmental intentions. Therefore, we added a behavioral measure by allowing participants to donate parts of their earnings to environmental organizations.

## Study 4

We preregistered three main hypotheses: **H1**. Pro-environmental intentions and behavior are positively associated with self-reported kama muta, controlling for prior climate attitudes and other emotions; **H2**. Pro-environmental intentions and behavior will be stronger in the kama muta condition; **H3**. The relationship between the main manipulation and pro-environmental intentions or behavior is mediated by self-reported kama muta.

### Method

#### Participants

In Study 4, we primarily focused on the experimental effect of kama muta on intentions. This effect was rather small in Study 3 (*d =* 0.14) but we expected a stronger difference due to more carefully controlled manipulations of at least magnitude *d* = 0.30. Employing G*Power 3.0 at an alpha level of 0.05, a power of 0.90, and a one-tailed test suggested a final sample size of 382. Considering possible exclusions, we pre-registered our final sample size at 400.

A total of 400 participants were recruited through the crowdsourcing website Prolific.ac requesting participants with US nationality. Based on pre-registered exclusion criteria, 15 participants were excluded from this study as they did not attempt at least 50% of the questionnaire (0), spent less than 3 min on the total survey (0), spent less than 90% of the time of the video on the page presenting the video (15), and/or failed an attention check question (0). The final sample size consisted of 385 participants (181 females, 202 males, and 2 other), aged 18 to 73 years (*M* = 36.27, SD = 11.98). The majority indicated US American as their nationality (379). Participants received $2.60 for participating in the study and a possible bonus payment of up to $1.

#### Design

We employed a between-subject design with participants being randomly allocated to watch either a *moving* (*n =* 183) or *neutral* (*n =* 202) video clip about climate change. The moving video was the same as in Study 1, featuring the narrative that humans might overcome climate change by acting together, and was narrated by Morgan Freeman (“Make a World of Difference”). The control video clip was from National Geographic including factual information about climate change. We edited the video by recording a more neutral voiceover and adding more neutral background music (see Supplementary material).

#### Measures

##### Climate attitudes and trait empathic concern

After providing informed consent and completing items about their demographic background (gender, age, nationality, number of children, and ownership of pet(s)), participants completed the 15 items of the Climate Change Attitude Survey as used in Study 2 (*α* = 0.93; Christensen and Knezek, 2015) and the seven items of the trait empathic concern (EC) subscale of the IRI (*α* = 0.88) as in Study 2. To shift the focus away from their attitudes toward climate change, participants then completed five filler items, two involving the sorting of odd or even numbers/words, two trivia questions, and one attention check. Participants were then shown one of the two videos and we recorded the amount of time they spent on the page with a timer.

##### Kama muta and other emotions

Participants then completed the same kama muta (three items each on sensations and labels; *α* = 0.87), anger (*α* = 0.90), fear (*α* = 0.88), and sadness (*α* = 0.83) items as in the previous studies. We added three items to assess *hope* (*α* = 0.92; *hopeful, optimistic, encouraged*) based on the modified differential emotion scale (Fredrickson et al., 2003) and one item asking how manipulated participants felt by the video. We then included the same four communal sharing appraisal items as in the previous studies (*α* = 0.95). All of the emotion and communal sharing items were completed on a 7-point scale from 0 (*not at all*) to 6 (*very much*). If participants indicated that they felt angrier or more moved/touched (defined by choosing a scale point of 3 or higher), they were asked to briefly write about what angered them or made them feel moved/touched.

##### Intentions and behavior

Afterward, participants completed the same Climate Intention Scale as in Study 3 (*α* = 0.94; general intentions *α* = 0.91; sharing with others *α* = 0.94) and were presented with a donation possibility. Specifically, participants were told that they would receive a bonus payment of $1 for participating in the study and that they could choose to donate as much of this as they wanted to one out of four non-governmental organizations. It was explained that participants might choose to donate all or none of the money without consequences for their participation in the study. We pre-selected four non-governmental organizations for this study: World Wildlife Fund (WWF), Rainforest Alliance, National Resources Defense Council (NRDC), and Amnesty International. All NGOs were briefly described, and we included a link to their main website for further information. We decided to include three NGOs mainly focusing on environmental topics and one NGO focusing on human rights to explore the impact of domain-related donations. We told participants that we would make donations after the study, and they had the opportunity to leave their email addresses in a different unrelated survey so that we could send them an email with an overview of the final amount of money donated and proof of the receipts.

Most participants (*n =* 239, 62.08%) donated at least some part of their bonus, while 142 decided not to donate any of their bonus (*M =* $0.38, SD *=* $0.40). Four participants failed to complete the question and according to our instructions, we donated the full amount on their behalf. Most participants selected Rainforest Alliance (*n =* 84; *M =* $0.65, SD *=* 0.33), followed by WWF (*n =* 72; *M =* $0.53, SD *=* 0.35), NRDC (*n =* 48, *M =* $0.63, SD *=* 0.34), and Amnesty International (*n =* 35, *M =* $0.62, SD *=* 0.35). In total, we donated $148.39 to the four NGOs.

### Results

#### Confirmatory results

As registered, reading time of the charity descriptions was log-transformed. An overview of the correlations among the main variables is provided in Supplementary Table S11; Figure 1.

First, we tested whether the experimental video indeed evoked more self-reported kama muta (registered manipulation check). We conducted two Welch’s *t*-tests with condition (−0.5 = control, 0.5 = kama muta video) as the predictor and self-reported kama muta feelings and sensations and self-reported communal sharing intensifications as outcomes. As expected, the kama muta video evoked more self-reported kama muta feelings (*M =* 1.70, SD *=* 1.27) and appraisals (*M =* 2.66, SD *=* 1.57) than the control video (feelings: *M =* 0.48, SD *=* 1.23, *t*(376.8) = 9.52, *p* < 0.001, *d =* 0.97 [0.76, 1.18]; CS: *M =* 0.98, SD *=* 1.49, *t*(374.4) = 10.72, *p* < 0.001, *d =* 1.10 [0.88, 1.31]).

For the first main hypothesis (H1), we performed three regression models adding 1) environmental intentions, 2) actual donation behavior, and 3) reading time (log-transformed) as outcomes. As predictors, we added self-reported kama muta, prior climate attitudes, and the other emotional reactions: self-reported anger, fear, sadness, and hope. For the intentions measure, we observed that kama muta positively predicted intentions when controlling for climate attitudes and other emotional experiences, replicating Studies 2 and 3 (see Table 3). The effect was strongest for kama muta when compared to all other emotions. Except for sadness all emotional experiences significantly and positively predicted pro-environmental intentions. Additional analyses for different types of intentions are presented in the (Supplementary Table S5).

Considering donation behavior (focusing only on no donations or donations toward pro-environmental organizations), we found no significant association with kama muta when controlling for climate attitudes and other emotions (see Table 5). Climate attitudes and hope turned out to be the only significant predictors of actual donation behavior. However, when considered on its own, kama muta positively predicted donation behavior (*r =* 0.10 [−0.003, 0.20]), although this effect was not statistically significant.

**Table 5:** Regression results using donation behavior (1) and reading time (2) as the criteria in Study 4.

Predictor	*b*	*b* 95% CI	*β*	*β* 95% CI	*sr* ^2^	*sr*^2^ 95% CI	*r*	Fit
*Donation Behavior*								
(Intercept)	−0.23	[−0.48, 0.01]						
Attitude	0.11^**^	[0.05, 0.18]	0.19	[0.08, 0.29]	0.03	[−0.00, 0.06]	0.26^**^	
Kama Muta	−0.04	[−0.08, 0.01]	−0.13	[−0.28, 0.03]	0.01	[−0.01, 0.02]	0.10	
Anger	0.01	[−0.02, 0.05]	0.06	[−0.09, 0.20]	0.00	[−0.01, 0.01]	0.11^**^	
Sadness	−0.00	[−0.05, 0.04]	−0.02	[−0.20, 0.16]	0.00	[−0.00, 0.00]	0.13^**^	
Anxiety	0.03	[−0.01, 0.07]	0.12	[−0.05, 0.30]	0.00	[−0.01, 0.02]	0.17^**^	
Hope	0.05^*^	[0.01, 0.08]	0.20	[0.05, 0.35]	0.02	[−0.01, 0.04]	0.16^**^	
								*R*^2^ = 0.09^**^
								95% CI [0.03,0.14]
*Reading Time (log)*								
(Intercept)	1.98^**^	[1.34, 2.62]						
Attitude	0.12	[−0.05, 0.29]	0.08	[−0.03, 0.19]	0.01	[−0.01, 0.02]	0.11^*^	
Kama Muta	−0.13*	[−0.24, −0.01]	−0.17	[−0.33, −0.02]	0.01	[−0.01, 0.03]	−0.01	
Anger	−0.09	[−0.19, 0.00]	−0.14	[−0.29, 0.00]	0.01	[−0.01, 0.03]	−0.10	
Sadness	0.05	[−0.07, 0.17]	0.08	[−0.11, 0.26]	0.00	[−0.01, 0.01]	−0.02	
Anxiety	0.03	[−0.08, 0.14]	0.04	[−0.13, 0.22]	0.00	[−0.00, 0.01]	−0.02	
Hope	0.13^**^	[0.03, 0.22]	0.21	[0.06, 0.37]	0.02	[−0.01, 0.04]	0.11^*^	
								*R*^2^ = 0.046^**^
								95% CI [0.00,0.08]

A significant *b*-weight indicates the *β*-weight and semi-partial correlation are also significant. *b* represents unstandardized regression weights. *β* indicates the standardized regression weights. *sr*^2^ represents the semi-partial correlation squared. *r* represents the zero-order correlation. The lower and upper limits of the confidence intervals are indicated in brackets. ^*^Indicates *p* < 0.05. ^**^Indicates *p* < 0.01.

Focusing on reading time, we found a significant association with kama muta when controlling for the other variables though in the opposite direction as expected (see Table 5). Self-reported kama muta negatively predicted reading time controlling for the other variables. Apart from kama muta, only hope showed a significant positive prediction. The zero-order correlation between kama muta and reading time suggested no significant effect (*r* = −0.009 [−0.11, 0.09]). When inspecting correlations among the predictors, we observed that kama muta and hope were strongly correlated (*r =* 0.71 [0.66, 0.76]). Exploratorily repeating the model without hope suggested no significant effect of kama muta, but a negative prediction by anger (see Supplementary Table S13).

Testing H2, we performed three Welch’s *t*-tests with 1) intentions, 2) donation behavior, and 3) reading time as outcomes and condition as the predictor. None of these measures differed significantly across the two videos. Intentions were higher after the moving video (*M =* 46.53, SD *=* 27.00) than after the neutral video (*M =* 45.28, SD *=* 27.40), but this difference was negligible (*d =* 0.05 [−0.15, 0.25]). Donations were higher after watching the neutral video (*M =* 0.39, SD *=* 0.41) compared to the moving video (*M =* 0.37, SD *=* 0.39), but this difference was again negligible (*d =* −0.04 [−0.25, 0.16]). Finally, reading time was higher after watching the moving video (*M =* 2.68, SD *=* 1.01) compared to the neutral video (*M =* 2.56, SD *=* 1.02), but this effect was again rather small (*d =* 0.12 [−0.08, 0.32]).

Focusing on H3, we tested mediation models using the three intention/behavior measures as outcomes, condition as the predictor, and kama muta as the mediator. The relationship between condition and intentions was mediated by kama muta (indirect effect: *b =* 0.28, *B =* 15.15 [95% Bootstrap CI: 11.67, 19.24]). When adding the mediator, the direct effect became negative (direct effect: *b =* −0.26, *B =* −13.90 [95% Bootstrap CI: −18.58, −9.03]). Similarly, the relationship between condition and donations was mediated by kama muta (indirect effect: *b =* 0.06, *B =* 0.05 [95% Bootstrap CI: 0.01, 0.09]). Again, when including the mediator, the direct effect became negative (direct effect: *b =* −0.08, *B =* −0.06 [95% Bootstrap CI: −0.15, 0.02]). Finally, for reading time, we found no significant indirect effect by kama muta (indirect effect: *b =* −0.02, *B =* −0.04 [95% Bootstrap CI: −0.14, 0.06], see Supplementary Table S15).

#### Exploratory results

We repeated the mediation models using appraisals as the mediator. Appraisals mediated the relationship between condition and pro-environmental intentions (indirect effect: *b =* 0.24, *B =* 13.22 [95% Bootstrap CI: 9.94, 17.29]), but not the relation between condition and donations (indirect effect: *b =* 0.01, *B =* 0.008 [95% Bootstrap CI: −0.04, 0.05]). However, we found a negative indirect effect between condition and reading time (indirect effect: *b =* −0.07, *B =* −0.14 [95% Bootstrap CI: −0.26, −0.03]), suggesting that the moving video increased communal sharing appraisals, which in turn was associated with less reading time of the descriptions about the environmental organizations (see Supplementary Table S15). We repeated the same mediation model with the item of whether participants felt manipulated as a mediator. We did not observe a statistically significant indirect effect of condition on pro-environmental intentions *via* feeling manipulated (indirect effect: *b =* 0.005, *B* = 0.27 [95% Bootstrap CI: −0.97, 1.66]), neither for donation behavior (indirect effect: *b =* 0.003, *B* = 0.002 [95% Bootstrap CI: −0.01, 0.02]) nor time spent (indirect effect: *b =* 0.002, *B* = 0.004 [95% Bootstrap CI: −0.01, 0.04], Supplementary Table S15). Feeling manipulated was associated with decreased pro-environmental intentions (*r* = −0.24 [−0.33, −0.14]), donation behavior (*r* = −0.15 [−0.25, −0.05]), and time spent (*r* = −0.09 [−0.19, 0.009]), but not substantially with kama muta (*r* = −0.009 [−0.11, 0.09]).

As in Study 2, but not Study 3, the association between kama muta ratings and intentions to share the information with others (*r* = 0.54 [0.46, 0.60]) was slightly stronger than with more general intentions (*r* = 0.47 [0.39, 0.55]).

Finally, we explored a possible moderation by climate attitudes but did not find any statistically significant effects for the relationships between kama muta and intentions, donation behavior, or reading time (see Supplementary Figures S3, S4). Similarly, we tested a possible moderation by trait empathic concern, but found only a small statistically significant effect for the relation between kama muta and pro-environmental intentions, suggesting the kama muta effect to be a bit smaller for high empathic concern (see Supplementary Table S14).

## Meta-analyses across all studies

**Figure 2:**
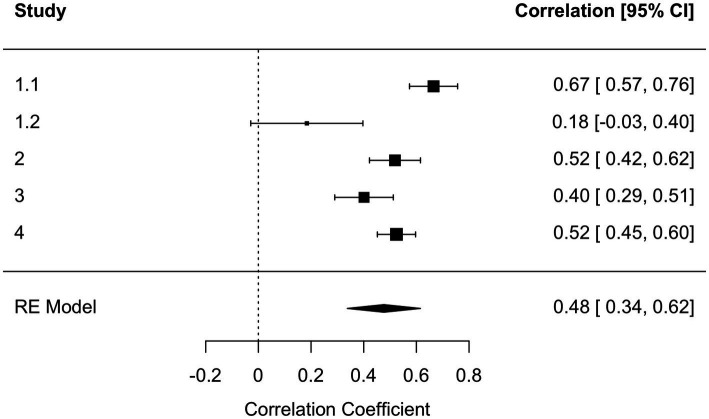
Forest plot of the random effects meta-analysis between kama muta and intentions across Studies 1 to 3. *Q*(4) = 23.66, *p* < 0.001, *I*^2^ = 88.83 [64.73, 98.97].

**Figure 3:**
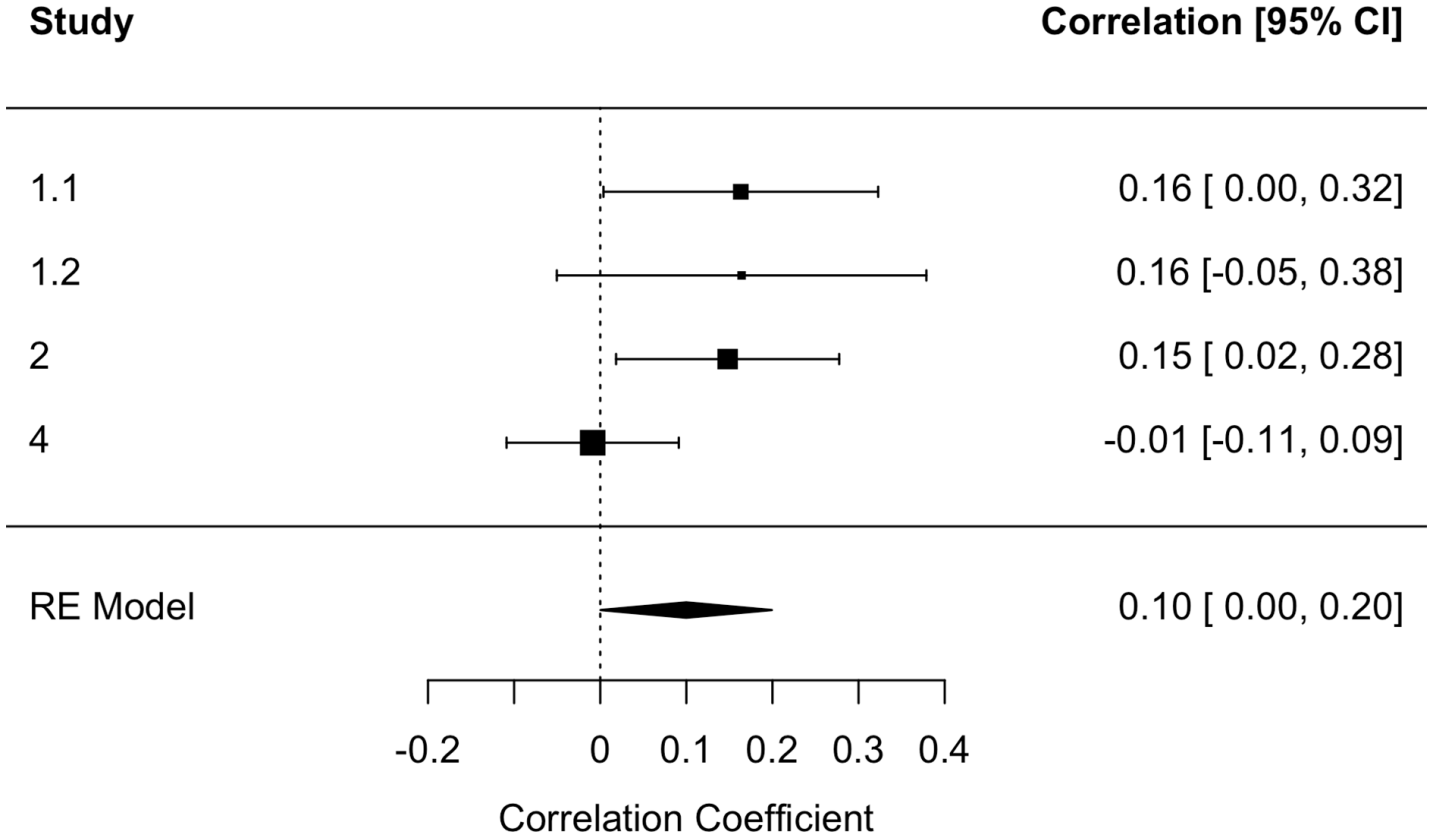
Forest plot of random effects meta-analysis between reading time (log) and kama muta across Studies 1 and 2. *Q*(3) = 5.72, *p =* 0.126, *I*^2^ = 48.29 [0, 94.75]. Note that the lower confidence interval is 0.0004.

**Figure 4:**
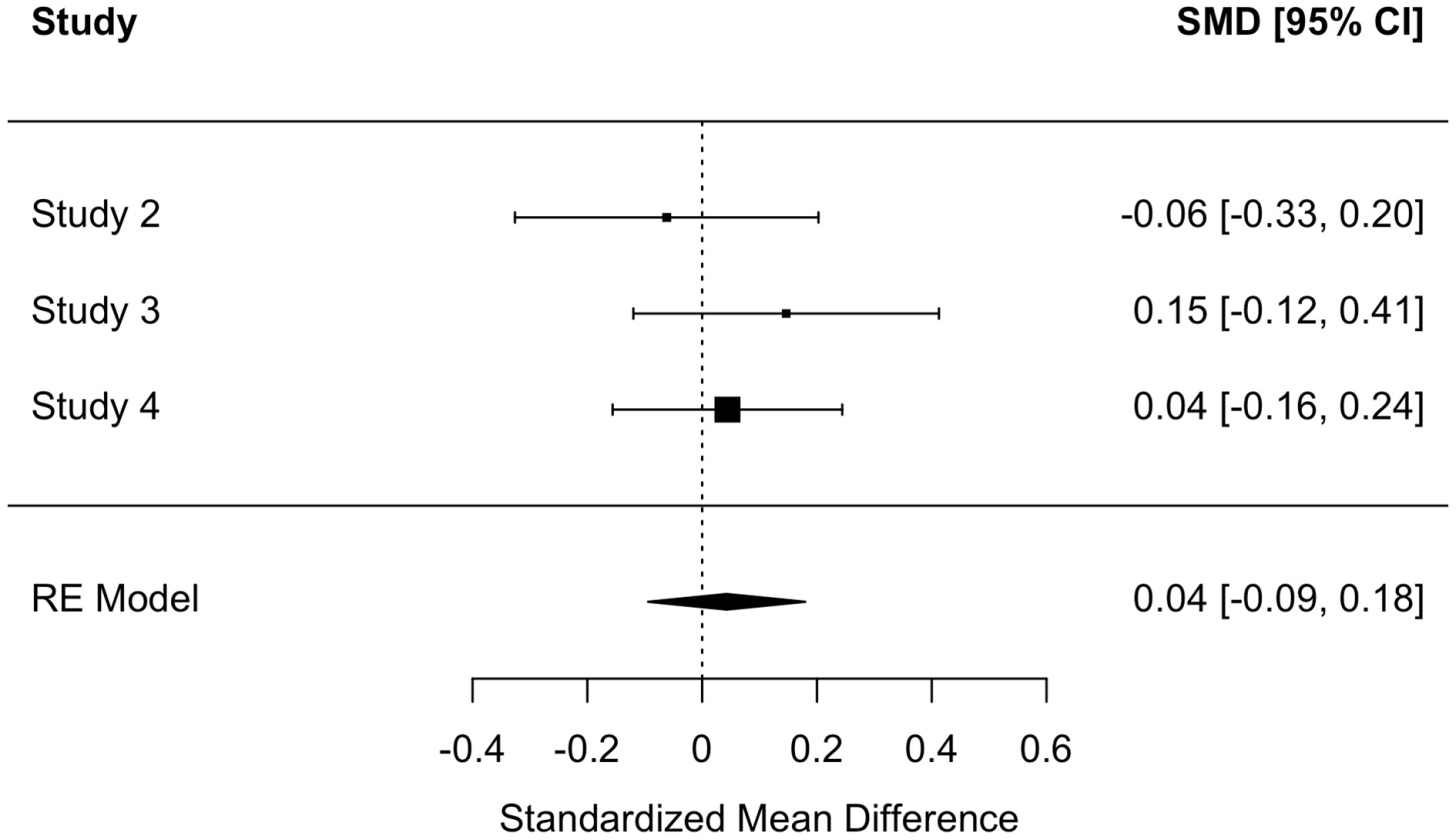
Forest plot of random effects meta-analysis of pro-environmental intentions between conditions (personal vs. neutral) across Studies 2, 3, and 4. *Q*(2) = 1.18, *p =* 0.554, *I*^2^ = 0 [0, 96.41].

To get a systematic overview of the effect of kama muta on intentions, we computed four internal meta-analyses (see Goh et al., 2016): one on the relationship between self-reported kama muta and intentions, the second between self-reported kama muta and the log-transformed reading time, the third for intentions as a function of experimental condition, and the fourth on the indirect effect of kama muta on the relation between the experimental condition and intentions. We computed three random effect models with a REML estimation in *metafor* (Viechtbauer, 2010). For the indirect effect, we employed the *metaSEM* package (Cheung, 2015). For the relation between kama muta and intentions, we found a meta-analytical effect size of *r =* 0.48 [0.34, 0.62] (Figure 2). We observed some heterogeneity mostly based on a smaller effect for the Norwegian sample in Study 1. For the relation between kama muta and reading time, we found a smaller, but also positive association, *r* = 0.10 [0.0004, 0.20] (Figure 3). This association was fairly homogenous across the first three studies but differed in Study 4. We found a non-significant overall small effect, *d =* 0.04 [−0.09, 0.18], of the experimental manipulations on intentions that were quite homogenous across studies (Figure 4). While we found no direct effect of our manipulations, we observed a significant overall indirect effect *via* kama muta, *b* = 0.21 [0.14, 0.28] (Supplementary Figure S6).

### General discussion

In four studies, we investigated the role of emotions, and particularly that of kama muta, in willingness to act on climate change. Based on previous findings (e.g., Zickfeld et al., 2017; Seibt et al., 2019; Landmann and Rohmann, 2020; Grüning and Schubert, 2022), we hypothesized that media content focusing on others in need or highlighting common fates could evoke feelings of kama muta, which in turn would be associated with one’s motivation to act on climate change. The results of four studies confirmed that emotions elicited through media content were positively associated with pro-environmental attitudes and intentions. More specifically, feelings of kama muta evoked through environmental media content were associated with climate-action intentions in all studies, also when controlling for prior attitudes and feelings of anger, anxiety, and sadness.

In addition, we found that the conditions designed to evoke more kama muta indeed did so, as indicated by higher values on the short form of the KAMMUS Two scale. We used the sensation and label subscales as one index and the appraisal subscale as another, and found parallel effects on both, suggesting that we indeed evoked kama muta through the media content presented. These findings suggest that environmental media content indeed has the potential of evoking feelings of kama muta. However, we found no causal effect of kama muta on pro-environmental intentions, but the effect of condition on intentions was only indirect, that is mediated through experienced kama muta (see Diessner et al., 2022).

Although all four studies showed associations between evoked kama muta and intentions to act on climate change, results were less consistent when considering the association between kama muta and actual pro-environmental actions. Studies 1 and 2 suggested that feelings of kama muta weakly predicted reading time on a page presenting pro-environmental information. Similarly, Study 4 found a small association between kama muta and donation behavior, though not statistically significant.

What are the practical implications of these results? To find out, we will first discuss the measurement choices we made regarding intentions and actions, then discuss the meaning of the indirect effects, and finally, compare kama muta to other emotions we measured.

### Climate change mitigation: intentions and behavior

In Study 1, we measured pro-environmental intentions broadly, not just related to climate change, while we developed and refined an intention measure specific to climate change for Studies 2–4. The behaviors we focused on as our main outcome variables were information sharing, climate activism, information seeking, and lifestyle choices. We measured information sharing through intentions to post on social media, discuss the message with others, and similar items. We measured climate activism through intentions to support or participate in climate movements and organizations, as well as through donations to such organizations (Study 4). Information seeking was assessed through intentions to find out more, as well as through reading time. Lifestyle choices that limit energy, resource, and greenhouse-gas-intensive food demand were assessed through intentions for impactful actions.

We found the four intention subscales to be internally consistent, and to form a consistent general intention scale. This suggests that the four types of motivation are closely related in participants’ minds. Kama muta showed significant, medium associations with the combined intention measure in all four studies, with a meta-analytical effect size of *r =* 0.48. It showed a small, positive, and significant meta-analytical association with reading time, *r* = 0.10, and a small, positive, non-significant correlation with donation behavior, *r =* 0.10, in Study 4. These effects were in line with predictions, but the associations with our behavioral measures were smaller than expected. We, therefore, did not power our studies adequately to detect such small effects (see Supplementary Figure S5; to detect an effect size of *r =* 0.10 with a power of 80% and an alpha of 0.05 requires at least 782 participants based on G^*^Power 3). Future studies should thus either increase the number of participants per study or increase the sensitivity of the measures, as discussed below.

While intentions to act do not automatically translate into actions, intentions typically precede actions (Ajzen, 1991). This is particularly the case for effortful actions such as changing one’s habit or incurring costs for an action. Thus, intentions often seem to be necessary but not sufficient conditions for actions (Sheeran, 2002). Strengthening goal intentions can thus be considered as a first step in a chain, motivating the action, while implementing it should follow once the goal intention is stronger than competing goals. Major barriers to acting on the intention then include not identifying a good opportunity to act and not having the resources or behavioral repertoire to act (Sheeran and Webb, 2016). These barriers are exacerbated by the existence of strong countervailing habits.

In the current studies, we found that kama muta evoked by media content related positively to intention to act even when controlling for prior climate change attitudes. We deliberately constructed a scale to measure intentions widely. It seems unlikely that a person would watch a video clip and then install solar panels on their roof as a direct consequence. Climate change mitigation is by its nature a collective endeavor, and social identity and norms are perhaps the most important determinants of individual behavior (McKenzie-Mohr, 2011). Thus, a complete model of attitude and behavior change through media has to take into account the social reverberations of the media content and the new ideas it inspires.

Kama muta is evoked by compassion (Zickfeld et al., 2017) and a sense of community during media exposure, and it is an emotion that people want to share with others (Fiske et al., 2019). While just sharing the emotion might not lead to any measurable action in the short term, repeated sharing, especially with a clear connection to a mitigation path, should lead to a community-level behavior change through changed social norms. Future research should test this extended model, based on the current findings.

Our model predicted kama muta to be associated with motivation, and with behavior through motivation.^8^ In line with this, we found much smaller correlations of kama muta with the behavioral measures for information seeking and activism, reading time, and donations, than for the intention measures. Behavior is influenced by many more factors than behavioral intentions, such as prior knowledge, prior donation behavior, habits, current time constraints, attitudes to these specific organizations, the alignment of the specific behavior chosen with the message content, the framing of the behavior (see, e.g., Hardisty et al., 2010), or current financial situation (Sheeran and Webb, 2016). How, then, does kama muta relate to action? We suggest that repeated kama muta, especially when shared in a community, increases the likelihood of climate action for actions that are closely related to the message content, and that are easy, accessible, and cheap. The work for environmental protection task (Lange and Dewitte, 2022) is a new measure designed to assess such action tendencies, and future research should employ such measures, preferably in a longitudinal, repeated-measures design. This way, not only intention but also effort as another important aspect of motivation can be assessed as a function of kama muta, while minimizing the influence of other determinants of action.

### Indirect effect in the absence of a direct effect

Across the three experimental studies (Study 2–4), we did not observe a consistently significant causal effect of media selected to evoke kama muta on pro-environmental intentions or behavior. Some of the effects were too small to be properly detected with our sample sizes, other effects were in the opposite of the predicted direction. Therefore, it is unclear whether kama muta and pro-environmental intentions show a positive correlation because feelings of kama muta can increase pro-environmental intentions (and behavior), because pro-environmental intentions (and behavior) can increase feelings of kama muta, or due to a third unmeasured variable. As reviewed by Schneider et al. (2021), positive emotions might not only motivate pro-environmental intentions or behavior but engaging in pro-environmental behavior might also trigger positive emotions. Based on the kama muta theory, we expected that feelings of kama muta can increase motivations (and behavior) to engage in pro-environmental behavior if this behavior can be formulated in terms of communal sharing—pro-social behavior that benefits other individuals or the planet (Fiske et al., 2019). However, studies have found that engaging in pro-environmental behavior can increase feelings of *warm glow* (Taufik et al., 2015), which might be related to the *warm* feelings typically experienced in kama muta (Fiske et al., 2019), providing a potential explanation for a causal effect from intentions or behavior to kama muta. It is also possible that individuals reporting higher pro-environmental intentions also value these actions more (Steg et al., 2014) and pro-environmental values might cause increased feelings of kama muta for media focusing on environmental-related topics (or *being moved* in general, see Cova and Deonna, 2014; Landmann et al., 2019).

In our studies, we chose to control for climate attitudes rather than environmental values. Only in Study 1, we had an environmental value scale, namely the New Ecological Paradigm. Arguably, some of the items in the CCAS that we employed to measure climate attitudes in Studies 1–4 relate to values, such as “Knowing about environmental problems and issues is important to me.” We found no consistent moderation effect for these control variables on the relation between kama muta and intention. Nevertheless, future studies could experimentally manipulate pro-environmental values to see whether they increase feelings of kama muta.

In addition, it has been discussed that emotions typically show strong correlations with pro-environmental intentions, but only weak causal effects, just as in the present case, due to *affect generalization* (Landmann, 2020). Landmann argues that short-lasting emotional episodes, such as the ones elicited by video or audio clips, are most likely to affect intentions, also in the long run, if they can generalize from the emotional episode to a general feeling, called *chronic affect* or *affective attitudes* (see also Schwartz and Loewenstein, 2017; Landmann and Rohmann, 2020). Here, we mainly recorded participants’ self-reported appraisal sensations, and feelings of kama muta to the specific stimuli, but did not assess more general affective attitudes of kama muta. In addition, the use of repeated exposure to emotional stimuli might strengthen the effect on intentions over time. Our effect size across the three studies was considerably small (*d* = 0.04), and repeated exposure and a focus on general affective attitudes could potentially increase this effect.

In line with previous similar studies (Diessner et al., 2022), we found an indirect effect of our message types on intentions and actual behavior *via* evoked kama muta in the absence of a direct effect. This finding was thus not completely unexpected, but it is important to consider what it means for campaign design. One reason for such a pattern can be a confounder, which is an unmeasured common cause of evoked kama muta and the outcomes of intentions and behavior (Loeys et al., 2015). One obvious candidate for such a common cause is the individual’s prior attitude regarding climate change: Someone highly concerned about climate change may be both more easily moved by climate change messages and intend to do more about climate change. We did find the latter relationship. However, prior attitudes did not moderate the effect of kama muta on intentions consistently. Another potential common cause could be trait empathic concern, as prior research found this to be a trait predictive of kama muta (Zickfeld et al., 2017). Again, we observed in Studies 2 and 4 that participants high in trait empathic concern reported higher intentions to do something about climate change. However, we only found a small interaction effect in Study 4 by trait empathic concern.

Another reason can be the presence of a suppression effect, which is an unmeasured effect from the manipulation of the outcome through a mediator variable with the opposite sign of the measured effect (MacKinnon et al., 2000). This interpretation is corroborated by the finding that the direct effect of condition on intentions (and on reading time in Study 2) became negative when controlling for experienced kama muta.

We suspected that feeling manipulated could have such a suppression effect (Ettinger et al., 2021) but did not find evidence for that in Study 4. What else could be the suppressor? According to the theory of planned behavior, the control conditions in Studies 2–4 could have positively influenced the strength of behavior-outcome beliefs, the value of these outcomes (these together form the behavioral attitude), the subjective norm, or the perceived control. In Study 3, we used a control condition on trying something new for 30 days as a good way of forming new habits, which conceivably could have influenced perceived control over new behavior.^9^ The control conditions in Studies 2 and 4 focused to a larger extent than the moving condition on factual information about climate change and its consequences presented by authorities. This could have had a larger effect on some aspects of the attitude component. Future research should use a broader array of media messages and measure more predictors of intention, in addition to emotion measures.

### Other emotions

In each study, we ran additional analyses to test whether other emotions evoked by the media messages would also predict intentions and behavior when all assessed emotions, as well as prior climate attitudes, were concurrently used as predictors. First, it is noteworthy that in all studies, all emotion concepts measured had significant positive zero-order correlations with intentions, and that these were largest for kama muta. The largest correlations were found for climate attitudes (except in Study 4 where kama muta and prior attitudes predicted to a similar extent).

In multiple regressions, sadness never predicted intentions significantly, and anxiety only in Study 4. Anger showed small, significant, independent associations with intentions in Studies 2 and 4 but not in 1 and 3. We assessed hope only in Study 4, and it had a small independent correlation with intentions as well. In Study 2, we found in addition that sadness predicted reading time positively in the multiple regression. In Study 4, hope predicted reading time positively, and kama muta negatively in the multiple regression. Hope and climate attitudes were the only variables predicting donations in the multiple regression.

The patterns regarding intentions are similar across studies and largely consistent with hypotheses. The patterns for the behavioral outcomes are somewhat inconsistent and puzzling. For example, while the meta-analytic association of kama muta with reading time is positive and significant, the association of kama muta with reading time became significantly negative when controlling for prior attitudes and other emotions in Study 4.

It may help, then, to again conceptualize climate action as essentially collective action. Even if done individually, it has to be based on the hope that many others will act similarly, and potentially on the goal to convince others through words or actions because it can only have a tangible effect when done at scale. Building on the study by Landmann and Rohmann (2020), Lizarazo Pereira et al. (2022) recently found that kama muta toward the Black-lives-matter movement mediated between collective efficacy and collective action intentions. They found a parallel path from injustice perceptions through anger on intentions.

Collective efficacy is closely related to hope, in that it is about the belief that the movement together can bring about change (Braithwaite, 2004). Kama muta without hope is thus unlikely to lead to action, whereas a specific, action-oriented, and collectively shared type of hope gives rise to kama muta, which becomes a motivator for continued activism. Our studies were not specifically designed to test such a model. However, the independent effects of anger and hope with motivations in some of our studies make it worthwhile examining this proposition further, by specifying the object of the emotions and testing more complex models such as the mentioned parallel mediation model. For kama muta, the compassion and communal sharing with victims of climate change can be one object evoking the emotion, potentially more related to information sharing, while increased communal sharing with fellow activists (in the simple sense of taking pro-environmental action) is another object evoking the emotion, potentially more closely related to motivation for activism.

## Limitations

As with all studies, so did our studies have several limitations. First, we conducted the studies only in Norway and the United States, making generalization beyond these two countries difficult. Second, we did not have a truly neutral baseline condition in any of the studies, which does not allow us to draw strong conclusions about the absence of the experimental effect. Third, we did not measure the objects of the emotions, which would have allowed us to understand more about the concrete elicitors, nor did we measure other sources of intentions, which would have allowed us to examine the potential effects of the conditions on other predictors of intentions. Fourth, a longitudinal design with a pre-measure of intentions could have allowed us to examine changes in intentions more directly. Fifth, the use of a behavioral measure that assesses efforts toward climate change mitigation without being influenced by many external variables could have increased the intention-behavior consistency. Sixth, ideally, different emotions should be measured with the same level of detail (number of items), which we did not do. Finally, Studies 1–3 consisted of small samples that were based on sample size justifications due to rules of thumb or effect size conventions that have since been called into question (Lakens, 2022). The experimental effects on intentions and behavior were rather small and our samples were not adequately powered to detect such effects. The meta-analytic approach can somewhat redeem this shortcoming, but future studies would need to focus on more valid sample size justifications such as selecting the smallest effect of interest.

Furthermore, given how much people are exposed to media already, a single media exposure is unlikely to bring about lasting behavioral changes (Abraham et al., 2010; Landmann, 2020), particularly for behaviors with significance for a person’s identity, group belonging, and ideology. Even short-term motivational changes are difficult to achieve. For example, a study comparing fear-and hope-inducing videos about climate change with neutral ones found no effect of condition on willingness to act (Ettinger et al., 2021).

## Conclusion

Across four studies, we found an association between kama muta evoked by media messages and intentions for actions that can contribute to climate change mitigation. This effect remained significant when controlling for prior climate change attitudes, and for other emotional responses, such as sadness, anger, and anxiety. It was not moderated by prior climate change attitudes, meaning that being more moved by the media message predicted more intentions for climate skeptics as well as people seeing climate change as a real and serious problem needing urgent action. In three studies, we compared a more moving to a less moving message yet found only an indirect effect of condition on intention through evoked kama muta. We conclude that kama muta could motivate climate action even in persons not highly engaged already. However, before this effect can be used in media campaigns, it has to be better understood.

## Data availability statement

The datasets presented in this study can be found in online repositories. The names of the repository/repositories and accession number(s) can be found below: https://osf.io/fsb4n/.

## Ethics statement

The studies involving human participants were reviewed and approved by University of Oslo, Department of Psychology, Internal Ethics Committee. The patients/participants provided their written informed consent to participate in this study.

## Author contributions

BS and JZ conceived the research question, hypotheses, preregistered, and ran Study 4. BS preregistered, ran Studies 1 and 2, drafted the introduction, and general discussion sections. NØ and BS prepared, preregistered, and ran Study 3. JZ analyzed all data, drafted the reports of all studies, prepared tables and graphs, and conducted the meta-analysis. All authors contributed to the article and approved the submitted version.

## Funding

This research was supported by internal research grants from the Department of Psychology, University of Oslo.

## Conflict of interest

The authors declare that the research was conducted in the absence of any commercial or financial relationships that could be construed as a potential conflict of interest.

## Publisher’s note

All claims expressed in this article are solely those of the authors and do not necessarily represent those of their affiliated organizations, or those of the publisher, the editors and the reviewers. Any product that may be evaluated in this article, or claim that may be made by its manufacturer, is not guaranteed or endorsed by the publisher.

## Footnotes

^1^Zickfeld et al. (2019) observed a correlation of *r* = 0.55 between feelings of kama muta and motivations. We expected a smaller correlation with pro-environmental intentions, but mainly chose *r* = 0.20 due to general effect size recommendations. Note that these have been recently called into question. We performed a sensitivity analysis based on our sample sizes to remedy this shortcoming (see Supplementary Figure S5).

^2^We recorded clicking behavior in the US sample for only three participants.

^3^Note that this decision was not pre-registered, but considered a sensible addition. Analyses without controlling for country are presented in the Supplementary Tables S3, S4.

^4^Originally, we preregistered excluding participants if they failed an item testing comprehension of the audio clip. However, we realized that the questions were ambiguously worded and observed a high exclusion rate (final *N* = 165). The main analyses applying this exclusion filter were similar to not excluding these participants and the results can be found in Supplementary Table S7.

^5^By mistake, the possibility to participate only for educational purposes was not registered. Conversely, we registered two exclusion criteria that we did not measure systematically: reporting technical problems with the audio and answering the manipulation check question wrong. Two participants indicated in the comments that they experienced technical difficulties, but they were already excluded by one of the other exclusion criteria.

^6^To decrease the time necessary to take the survey, we shortened the attitude measure from Study 2 to use in Study 3. Our goal was to keep most of the intention subscale because we sought to control for prior attitudes relating to the usefulness vs. futility of personal action against climate change. Based on a reliability analysis of the data from Study 2, we removed two items with an item-total correlation of below 0.60 (CCAS 10 and 15). In addition, we removed some near-synonyms from the Belief subscale, namely items 1, 4, 5, and 8 (based on inter-correlations with the selected items of over 0.80). We thus created a scale with nine items, four from the Belief subscale and five from the intention subscale.

^7^The study was originally conducted as part of a graduate thesis (Østby, 2018) that focused on climate refugees. Therefore, it included measures not focal to the current purposes, but we present them for reasons of transparency.

^8^Note that this prediction is consistent with kama muta theory, and also with the Theory of Planned Behavior (Ajzen, 1991), where attitudes are the sum of all the relevant beliefs multiplied by the value given to these specific outcomes. Kama muta, by being a positive emotion, can increase the value of the expected outcomes of various pro-environmental behaviors, for example, by increasing how much a person cares about others.

^9^We had not thought of that confound when choosing the control condition but think in retrospect that this choice of a control video was unfortunate, because the video propagated a tool for implementing new habits, which at least some of our intention items tapped into.
