# Supplementary material for: Global heart warming: kama muta evoked by climate change messages is associated with intentions to mitigate climate change
Source: Front Psychol. 2023 Apr 28;14:1112910. doi: 10.3389/fpsyg.2023.1112910 (PMC10175856; doi:10.3389/fpsyg.2023.1112910)
Supplement: Supplementary file 1 [file Presentation_1.PDF]

Global Heart Warming: Climate Change Messages Move People to Act by Inducing Kama Muta

Supplement.

OVERALL ANALYSIS PLAN 2

STUDY 1 3

- TABLE S1 3
- TABLE S2 4
- TABLE S3 5
- TABLE S4 6
- TABLE S5 8

STUDY 2 9

- TABLE S6 9
- FIGURE S1 10
- TABLE S7 11
- ANALYSES WITH EMPATHIC CONCERN 12
- TABLE S8 12

STUDY 3 13

- TABLE S9 13
- FIGURE S2 14
- TABLE S10 15
- RESULTS ON ATTITUDES TOWARDS CLIMATE REFUGEES 15

STUDY 4 16

- TABLE S11 16
- TABLE S12 17
- TABLE S13 18
- ANALYSES WITH EMPATHIC CONCERN 18
- TABLE S14. 19
- FIGURE S3 20
- FIGURE S4 21

MEDIATION MODELS 22

- TABLE S15 22

SENSITIVITY POWER ANALYSES 25

- FIGURE S5. 25

META-ANALYSIS 26

- FIGURE S6. 26

STIMULI 27

- STUDY 1 27
- STUDY 2 27
- STUDY 3 27
- STUDY 4 27

REFERENCES 27

Overall analysis plan

All analyses were performed in R Studio (*version 4.2.1*, R Core Team, 2022). We employed the packages *dplyr* (*version 1.0.10*; Wickham et al., 2019), *tidyr* (*version 1.2.1;* Wickham et al., 2019), *janitor* (*version 2.1.0;* Firke, 2021), and *reshape2* (*version 1.4.4;* Wickham, 2017) for data recoding and transformations, *ggplot2* (*version 3.3.6*; Wickham, 2016), *ggpubr* (*version 0.4.0;* Kassambara & Kassambara, 2020), and *cowplot* (*version 1.1.1;* Wilke, 2019) for graphical visualization, *psych* (*version 2.2.5*; Revelle, 2017) for factor analyses, *sjPlot* (*version 2.8.11*; Lüdecke, 2018) and *apaTables* (*version 2.0.8;* Stanley & Spence, 2018) for analyses and plotting, *lavaan* (*version 0.6-12;* Rosseel, 2012) for conducting the mediation analyses, and *metafor (version 3.8-1;* Viechtbauer, 2010) for meta-analyses. When running mediation models we estimated confidence intervals using 10,000 bootstrap samples (Hayes, 2017).

Study 1

Table S1

Means, standard deviations, and correlations with confidence intervals in Study 1 in the Norwegian sample.

Variable	*M*	*SD*	1	2	3	4	5	6	7	8
1. Kama Muta	1.77	1.20								
2. Communal Sharing	2.28	1.49	.82** [.73, .88]							
3. Anxiety	1.30	1.44	.66** [.51, .77]	.61** [.46, .73]						
4. Anger	1.75	1.91	.50** [.31, .65]	.40** [.20, .57]	.34** [.13, .52]					
5. Intentions	3.29	0.79	.18 [-.04, .39]	.17 [-.05, .38]	.06 [-.17, .27]	.23* [.02, .43]				
6. Reading time	3.62	1.06	.16 [-.06, .37]	.16 [-.06, .37]	-.05 [-.27, .17]	.40** [.20, .57]	.39** [.18, .56]			
7. EINS	4.22	1.29	.12 [-.10, .33]	.12 [-.10, .33]	.01 [-.21, .23]	.10 [-.12, .32]	.33** [.11, .51]	.05 [-.17, .27]		
8. NEP	2.88	0.61	.26* [.04, .45]	.23* [.01, .42]	-.01 [-.23, .21]	.32** [.11, .50]	.54** [.36, .68]	.20 [-.03, .40]	.31** [.10, .50]	
9. Climate Attitude	5.28	1.22	.14 [-.08, .35]	.08 [-.15, .29]	-.03 [-.25, .19]	.23* [.01, .43]	.66** [.51, .77]	.25* [.03, .45]	.39** [.18, .56]	.53** [.35, .67]

Note. M and *SD* are used to represent mean and standard deviation, respectively. Values in square brackets indicate the 95% confidence interval for each correlation. The confidence interval is a plausible range of population correlations that could have caused the sample correlation (Cumming, 2014). * indicates $p < .05$. ** indicates $p < .01$.

Table S2

Means, standard deviations, and correlations with confidence intervals in Study 1 for the US sample.

Variable	*M*	*SD*	1	2	3	4	5	6	7	8	9
1. Kama Muta	2.84	1.75									
2. Communal Sharing	2.88	1.91	.89**<br>[.85, .92]								
3. Anxiety	1.56	1.84	.44**<br>[.29, .56]	.45**<br>[.31, .57]							
4. Anger	1.17	1.57	.36**<br>[.21, .49]	.37**<br>[.22, .50]	.60**<br>[.48, .70]						
5. Intentions	3.39	1.06	.67**<br>[.56, .75]	.65**<br>[.55, .74]	.32**<br>[.16, .46]	.30**<br>[.14, .44]					
6. Reading time	2.30	1.02	.16<br>[-.00, .32]	.17*<br>[.01, .33]	.13<br>[-.03, .29]	.14<br>[-.03, .29]	.23**<br>[.07, .38]				
7. EINS	4.12	1.52	.41**<br>[.27, .54]	.40**<br>[.26, .53]	.23**<br>[.07, .38]	.09<br>[-.08, .25]	.46**<br>[.32, .58]	.12<br>[-.05, .27]			
8. NEP	2.71	0.92	.43**<br>[.28, .55]	.44**<br>[.30, .56]	.20*<br>[.03, .35]	.17*<br>[.00, .32]	.62**<br>[.51, .71]	.15<br>[-.01, .31]	.44**<br>[.30, .56]		
9. CCAS	3.91	0.83	.41**<br>[.26, .54]	.41**<br>[.27, .54]	.12<br>[-.04, .28]	.14<br>[-.02, .30]	.68**<br>[.58, .76]	.19*<br>[.03, .35]	.33**<br>[.17, .47]	.76**<br>[.68, .82]	
10. Climate Attitude	5.23	1.77	.48**<br>[.35, .60]	.47**<br>[.33, .59]	.26**<br>[.10, .41]	.17*<br>[.00, .32]	.73**<br>[.65, .80]	.06<br>[-.10, .23]	.41**<br>[.27, .54]	.69**<br>[.59, .77]	.84**<br>[.79, .88]

Note. M and *SD* are used to represent mean and standard deviation, respectively. Values in square brackets indicate the 95% confidence interval for each correlation. The confidence interval is a plausible range of population correlations that could have caused the sample correlation (Cumming, 2014). * indicates $p < .05$. ** indicates $p < .01$.

Table S3

Regression results of intentions or reading time (log) on kama muta in Study 1 without country.

Predictor	*b*	*b* 95% CI [LL, UL]	*beta*	*beta* 95% CI [LL, UL]	sr^2	sr^2 95% CI [LL, UL]	*r*	Fit
Pro-environmental intentions								
(Intercept)	2.57**	[2.37, 2.76]						
Kama Muta	0.32**	[0.26, 0.39]	0.55	[0.43, 0.66]	.30	[.20, .38]	.55**	
								R^2 = .297** 95% CI[.20,.38]
Reading Time (log)								
(Intercept)	2.81**	[2.52, 3.10]						
Kama Muta	-0.02	[-0.12, 0.08]	-0.02	[-0.16, 0.11]	.00	[.00, .02]	-.02	
								R^2 = .001 95% CI[.00,.02]

Note. A significant *b*-weight indicates the beta-weight and semi-partial correlation are also significant. *b* represents unstandardized regression weights. *beta* indicates the standardized regression weights. sr^2 represents the semi-partial correlation squared. *r* represents the zero-order correlation. *LL* and *UL* indicate the lower and upper limits of a confidence interval, respectively.

* indicates $p < .05$. ** indicates $p < .01$.

Table S4

Regression results using Pro-Environmental Intention and Reading Time (log) as the criterion

Predictor	Pro-Environmental Intentions				Reading Time (log)			
	b	**95% CI**	**Fit**	**Difference**	***b***	**95% CI**	**Fit**	**Difference**
(Intercept)	0.95**	[0.56, 1.33]			1.93**	[1.17, 2.69]		
Climate Attitude	0.37**	[0.30, 0.45]			0.19*	[0.04, 0.34]		
KM	0.21*	[0.03, 0.40]			0.22	[-0.14, 0.57]		
Climate Attitude:KM	-0.01	[-0.04, 0.03]			-0.05	[-0.11, 0.01]		
			R^2 = .597**				R^2 = .028	
			95% CI[.51,.65]				95% CI[.00,.07]	
(Intercept)	0.94**	[0.55, 1.33]			1.85**	[1.20, 2.50]		
Climate Attitude	0.36**	[0.28, 0.44]			0.04	[-0.09, 0.17]		
KM	0.21*	[0.02, 0.39]			0.09	[-0.22, 0.40]		
USNO	0.09	[-0.09, 0.28]			1.40**	[1.10, 1.71]		
Climate Attitude:KM	-0.00	[-0.03, 0.03]			-0.00	[-0.05, 0.05]		
			R^2 = .599**	ΔR^2 = .002			R^2 = .292**	ΔR^2 = .264**
			95% CI[.51,.65]	95% CI[-.01, .01]			95% CI[.19,.37]	95% CI[.17, .36]
(Intercept)	1.53**	[1.04, 2.01]			1.92**	[1.15, 2.70]		
EINS	0.28**	[0.17, 0.40]			0.23*	[0.05, 0.42]		
KM	0.45**	[0.26, 0.65]			0.20	[-0.10, 0.50]		
EINS:KM	-0.04*	[-0.08, -0.00]			-0.06	[-0.12, 0.01]		
			R^2 = .379**				R^2 = .028	
			95% CI[.28,.46]				95% CI[.00,.07]	
(Intercept)	1.50**	[1.02, 1.99]			1.80**	[1.14, 2.47]		
EINS	0.27**	[0.14, 0.39]			0.06	[-0.11, 0.22]		
KM	0.45**	[0.26, 0.64]			0.14	[-0.12, 0.40]		
USNO	0.16	[-0.07, 0.39]			1.40**	[1.09, 1.70]		
EINS:KM	-0.04	[-0.08, 0.00]			-0.01	[-0.07, 0.05]		
			R^2 = .385**	ΔR^2 = .005			R^2 = .292**	ΔR^2 = .264**
			95% CI[.28,.46]	95% CI[-.01, .02]			95% CI[.19,.37]	95% CI[.17, .36]

Predictor	Pro-Environmental Intentions				Reading Time (log)			
	b	**95% CI**	**Fit**	**Difference**	***b***	**95% CI**	**Fit**	**Difference**
(Intercept)	0.91**	[0.44, 1.39]			1.87**	[1.07, 2.68]		
NEP	0.69**	[0.51, 0.87]			0.39*	[0.09, 0.70]		
KM	0.42**	[0.23, 0.60]			0.01	[-0.30, 0.33]		
NEP:KM	-0.07*	[-0.13, -0.01]			-0.03	[-0.14, 0.08]		
			R^2 = .496**				R^2 = .046*	
			95% CI[.40,.56]				95% CI[.00,.10]	
(Intercept)	0.91**	[0.44, 1.39]			1.93**	[1.24, 2.63]		
NEP	0.68**	[0.50, 0.87]			0.04	[-0.23, 0.32]		
KM	0.42**	[0.23, 0.60]			-0.05	[-0.32, 0.22]		
USNO	0.03	[-0.18, 0.24]			1.39**	[1.09, 1.70]		
NEP:KM	-0.07*	[-0.13, -0.00]			0.05	[-0.05, 0.14]		
			R^2 = .496**	ΔR^2 = .000			R^2 = .302**	ΔR^2 = .255**
			95% CI[.40,.56]	95% CI[-.00, .00]			95% CI[.19,.38]	95% CI[.16, .35]
(Intercept)	-0.24	[-1.00, 0.52]			1.77**	[0.58, 2.96]		
CCAS	0.73**	[0.52, 0.94]			0.09	[-0.24, 0.41]		
KM	0.47**	[0.19, 0.75]			-0.12	[-0.56, 0.32]		
CCAS:KM	-0.05	[-0.12, 0.02]			0.05	[-0.06, 0.16]		
			R^2 = .644**				R^2 = .051	
			95% CI[.54,.70]				95% CI[.00,.12]	

Note. A significant *b*-weight indicates the semi-partial correlation is also significant. *b* represents unstandardized regression weights. sr^2 represents the semi-partial correlation squared. In brackets are the lower and upper limits of the confidence intervals.
* indicates p < .05. ** indicates p < .01.

Table S5

Regression results on intentions controlling for anger and anxiousness.

Predictor	*b*	*b* 95% CI [LL, UL]	*beta*	*beta* 95% CI [LL, UL]	*r*	Fit
(Intercept)	0.99**	[0.70, 1.27]				
Climate Attitude	0.36**	[0.30, 0.42]	0.59	[0.50, 0.68]	.72**	
Kama Muta	0.19**	[0.12, 0.25]	0.31	[0.21, 0.42]	.55**	
Anxious	-0.02	[-0.08, 0.04]	-0.04	[-0.15, 0.06]	.26**	
Angry	0.04	[-0.01, 0.10]	0.07	[-0.02, 0.17]	.26**	
						R^2 = .601** 95% CI[.52,.66]
(Intercept)	1.29**	[0.96, 1.62]				
NEP	0.54**	[0.42, 0.66]	0.46	[0.35, 0.56]	.59**	
Kama Muta	0.22**	[0.16, 0.29]	0.38	[0.27, 0.50]	.55**	
Anxious	-0.01	[-0.08, 0.06]	-0.01	[-0.13, 0.11]	.26**	
Angry	0.03	[-0.04, 0.09]	0.05	[-0.06, 0.16]	.26**	
						R^2 = .487** 95% CI[.39,.55]
(Intercept)	0.12	[-0.40, 0.63]				
CCAS	0.63**	[0.49, 0.77]	0.49	[0.38, 0.60]	.68**	
Kama Muta	0.26**	[0.19, 0.33]	0.43	[0.31, 0.55]	.55**	
Anxious	0.02	[-0.05, 0.10]	0.04	[-0.09, 0.17]	.26**	
Angry	0.03	[-0.05, 0.12]	0.05	[-0.08, 0.18]	.26**	
						R^2 = .645** 95% CI[.54,.70]
(Intercept)	1.87**	[1.55, 2.19]				
EINS	0.19**	[0.12, 0.27]	0.28	[0.17, 0.39]	.42**	
Kama Muta	0.27**	[0.19, 0.34]	0.45	[0.33, 0.58]	.55**	
Anxious	-0.04	[-0.11, 0.04]	-0.07	[-0.20, 0.06]	.26**	
Angry	0.07*	[0.00, 0.14]	0.12	[0.00, 0.24]	.26**	
						R^2 = .379** 95% CI[.27,.45]

Note. A significant *b*-weight indicates the beta-weight and semi-partial correlation are also significant. *b* represents unstandardized regression weights. *beta* indicates the standardized regression weights. sr^2 represents the semi-partial correlation squared. *r* represents the zero-order correlation. *LL* and *UL* indicate the lower and upper limits of a confidence interval, respectively.
* indicates $p < .05$. ** indicates $p < .01$.

Study 2

Table S6

Means, standard deviations, and correlations with confidence intervals in Study 2.

Variable	*M*	*SD*	1	2	3	4	5	6	7	8	9	10	11	12	13
1. Condition (0: neutral, 1: personal)	0.51	0.50													
2. Kama Muta	2.38	1.57	.25**<br>[.13, .37]												
3. Communal Sharing	2.10	1.72	.33**<br>[.21, .45]	.59**<br>[.50, .67]											
4. Anger	1.39	1.61	-.05<br>[-.18, .09]	.49**<br>[.38, .58]	.28**<br>[.16, .40]										
5. Anxiety	2.05	1.76	.01<br>[-.12, .14]	.63**<br>[.54, .70]	.35**<br>[.23, .46]	.64**<br>[.55, .71]									
6. Sadness	2.88	1.56	.11<br>[-.02, .24]	.63**<br>[.55, .71]	.29**<br>[.16, .41]	.58**<br>[.48, .66]	.69**<br>[.62, .76]								
7. Intentions	3.04	0.97	-.03<br>[-.17, .10]	.52**<br>[.41, .61]	.34**<br>[.22, .46]	.48**<br>[.37, .57]	.46**<br>[.34, .55]	.39**<br>[.27, .50]							
8. Intention Act	3.39	1.10	.02<br>[-.11, .15]	.32**<br>[.20, .43]	.21**<br>[.08, .33]	.31**<br>[.18, .42]	.32**<br>[.20, .44]	.27**<br>[.14, .39]	.83**<br>[.78, .87]						
9. Intention Do	3.84	0.94	.07<br>[-.06, .20]	.35**<br>[.22, .46]	.21**<br>[.08, .34]	.22**<br>[.10, .35]	.27**<br>[.15, .39]	.25**<br>[.13, .37]	.73**<br>[.66, .79]	.66**<br>[.58, .73]					
10. Intention Seek	3.03	1.24	-.10<br>[-.23, .03]	.44**<br>[.33, .54]	.27**<br>[.14, .39]	.47**<br>[.36, .57]	.40**<br>[.29, .51]	.38**<br>[.27, .49]	.92**<br>[.90, .94]	.67**<br>[.60, .74]	.60**<br>[.50, .68]				
11. Intention Share	2.23	1.21	-.04<br>[-.17, .09]	.58**<br>[.48, .66]	.42**<br>[.30, .52]	.51**<br>[.40, .60]	.47**<br>[.36, .57]	.36**<br>[.24, .47]	.85**<br>[.80, .88]	.52**<br>[.42, .61]	.42**<br>[.30, .52]	.74**<br>[.67, .79]			
12. Reading time	4.22	0.95	-.03<br>[-.16, .10]	.15*<br>[.02, .27]	-.02<br>[-.15, .11]	.12<br>[-.01, .25]	.09<br>[-.04, .22]	.22**<br>[.09, .35]	.31**<br>[.19, .43]	.20**<br>[.07, .33]	.28**<br>[.15, .40]	.35**<br>[.23, .46]	.22**<br>[.09, .34]		
13. CCAS	4.04	0.83	.06<br>[-.07, .19]	.17*<br>[.04, .30]	.05<br>[-.09, .18]	.10<br>[-.04, .23]	.14*<br>[.01, .27]	.21**<br>[.08, .33]	.57**<br>[.48, .66]	.69**<br>[.61, .75]	.63**<br>[.54, .70]	.48**<br>[.37, .57]	.27**<br>[.14, .39]	.21**<br>[.08, .33]	
14. EC	3.79	1.04	.11<br>[-.02, .24]	.40**<br>[.28, .50]	.23**<br>[.10, .35]	.16*<br>[.03, .29]	.18**<br>[.05, .31]	.33**<br>[.21, .44]	.40**<br>[.29, .51]	.40**<br>[.29, .51]	.37**<br>[.24, .47]	.38**<br>[.26, .49]	.24**<br>[.12, .36]	.19**<br>[.06, .32]	.31**<br>[.18, .42]

Note. M and *SD* are used to represent mean and standard deviation, respectively. Values in square brackets indicate the 95% confidence interval for each correlation. The confidence interval is a plausible range of population correlations that could have caused the sample correlation (Cumming, 2014). * indicates $p < .05$. ** indicates $p < .01$.

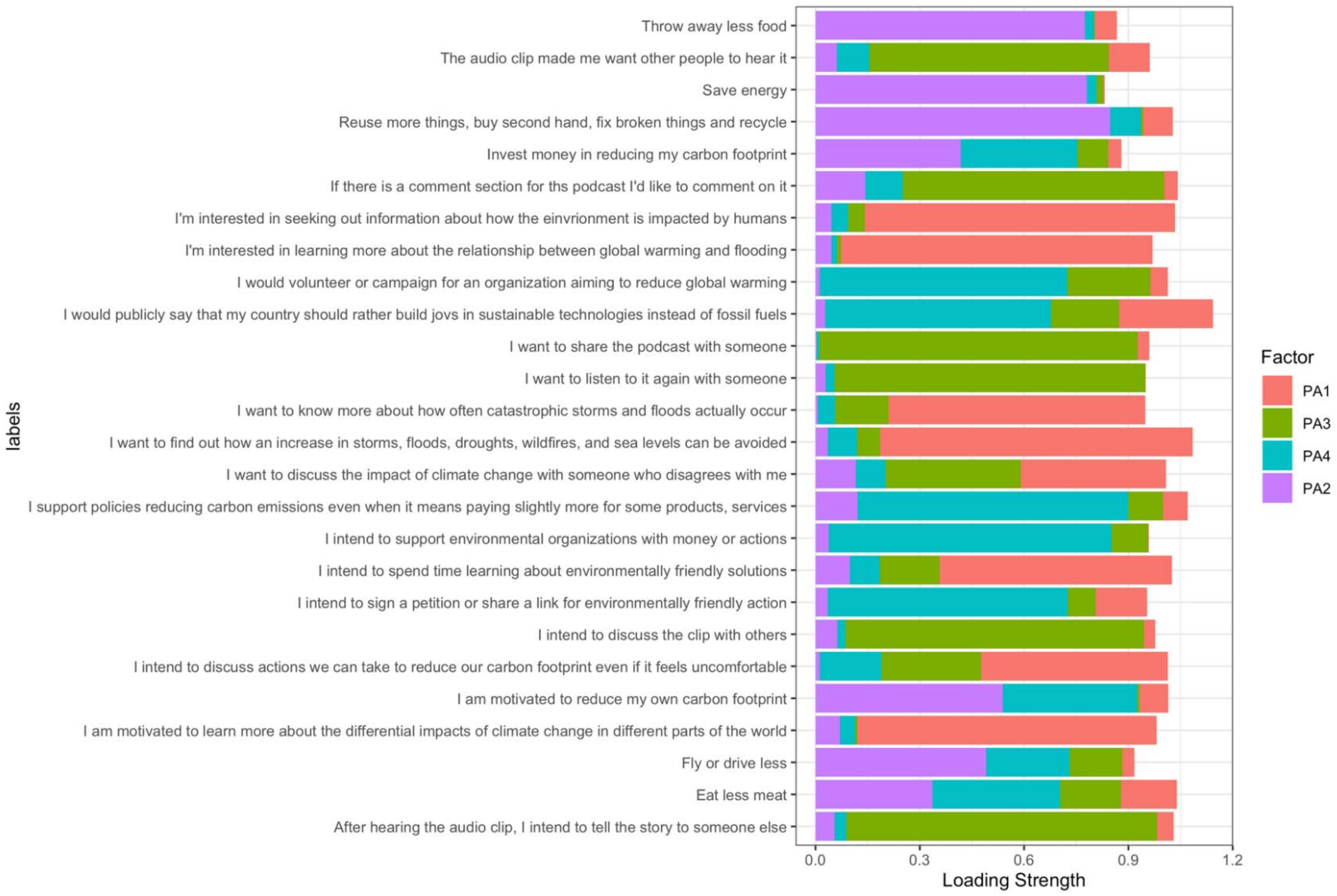

Figure S1. Overview of factor loadings for the pro-environmental intention items in Study 2. Parallel analysis suggested 4 factors.

Table S7

Regression results for the main models applying comprehension filter.

Predictor	*b*	*b* 95% CI [LL, UL]	*beta*	*beta* 95% CI [LL, UL]	*r*	Fit
Pro-Environmental Intentions						
(Intercept)	-0.09	[-1.03, 0.17]				
CCAS	0.62**	[0.48, 0.76]	0.47	[0.37, 0.58]	.53**	
Kama Muta	0.21**	[0.13, 0.29]	0.36	[0.21, 0.50]	.52**	
Anger	0.20**	[0.11, 0.28]	0.33	[0.19, 0.48]	.48**	
Sadness	-0.08	[-0.17, 0.02]	-0.13	[-0.28, 0.03]	.38**	
Anxiety	0.01	[-0.08, 0.10]	0.02	[-0.14, 0.19]	.41**	
						R^2 = .554**<br>95% CI[.44,.62]
Reading Time (log)						
(Intercept)	3.04**	[2.17, 3.91]				
CCAS	0.20	[-0.00, 0.41]	0.15	[-0.00, 0.30]	.19*	
Kama Muta	0.02	[-0.10, 0.15]	0.04	[-0.16, 0.24]	.14	
Anger	0.02	[-0.11, 0.14]	0.03	[-0.18, 0.23]	.10	
Sadness	0.16*	[0.02, 0.31]	0.26	[0.04, 0.49]	.21**	
Anxiety	-0.10	[-0.23, 0.04]	-0.17	[-0.41, 0.07]	.07	
						R^2 = .081*<br>95% CI[.00,.15]

Note. A significant *b*-weight indicates the beta-weight and semi-partial correlation are also significant. *b* represents unstandardized regression weights. *beta* indicates the standardized regression weights. sr^2 represents the semi-partial correlation squared. *r* represents the zero-order correlation. *LL* and *UL* indicate the lower and upper limits of a confidence interval, respectively. * indicates $p < .05$. ** indicates $p < .01$.

Analyses with Empathic Concern

We observed a positive correlation between kama muta ratings and trait empathic concern (r = .40 [.28, .50]). In addition, trait empathic concern correlated positively with intentions (r = .40 [.29, .51]) and reading time (log) (r = .19 [.06, .32]).

We also explored the interaction between kama muta ratings and trait empathic concern on intentions and reading time but did not observe a statistically significant interaction. Intentions and reading time were strongest for participants experiencing strong kama muta and scoring high on trait empathic concern.

Table S8

	**Intention**				
Predictors	*Estimates*	*std. Beta*	*CI*	*standardized CI*	*p*
(Intercept)	3.02	-0.02	2.90 – 3.13	-0.14 – 0.09	**<0.001**
EC	0.24	0.26	0.12 – 0.36	0.13 – 0.39	**<0.001**
KM	0.26	0.42	0.18 – 0.33	0.30 – 0.54	**<0.001**
EC* KM	0.04	0.06	-0.03 – 0.10	-0.05 – 0.17	0.263
Observations	220				
R^2 / R^2 adjusted	0.320 / 0.310				
	**Reading time**				
Predictors	*Estimates*	*std. Beta*	*CI*	*standardized CI*	*p*
(Intercept)	4.20	-0.02	4.06 – 4.33	-0.16 – 0.12	**<0.001**
EC	0.16	0.18	0.03 – 0.30	0.03 – 0.33	**0.020**
KM	0.05	0.08	-0.04 – 0.14	-0.06 – 0.22	0.266
EC * KM	0.03	0.06	-0.04 – 0.11	-0.07 – 0.19	0.375
Observations	220				
R^2 / R^2 adjusted	0.046 / 0.033				

Note: EC = Empathic Concern, KM = Kama Muta.

Study 3

Table S9

Means, standard deviations, and correlations with confidence intervals in Study 3.

Variable	*M*	*SD*	1	2	3	4	5	6	7	8	9
1. Condition (0: neutral, 1: personal)	0.45	0.50									
2. Kama Muta	1.92	1.32	.47**								
			[.36, .57]								
3. Communal Sharing	1.82	1.54	.54**	.60**							
			[.43, .62]	[.51, .68]							
4. Anger	0.73	1.10	.61**	.47**	.42**						
			[.52, .69]	[.36, .57]	[.30, .52]						
5. Anxiety	1.12	1.41	.65**	.59**	.51**	.65**					
			[.56, .72]	[.50, .67]	[.41, .60]	[.57, .72]					
6. Sadness	1.46	1.53	.75**	.59**	.50**	.74**	.80**				
			[.68, .80]	[.50, .67]	[.40, .60]	[.67, .79]	[.74, .84]				
7. Intention	44.48	21.81	.07	.40**	.19**	.28**	.23**	.25**			
			[-.06, .20]	[.28, .51]	[.06, .32]	[.16, .40]	[.10, .35]	[.12, .37]			
8. Intention General	31.49	26.82	-.08	.37**	.19**	.13	.08	.08	.74**		
			[-.21, .06]	[.25, .48]	[.06, .32]	[-.00, .26]	[-.06, .21]	[-.06, .21]	[.67, .79]		
9. Intention Share	50.05	23.98	.13	.34**	.16*	.30**	.26**	.29**	.95**	.48**	
			[-.00, .26]	[.22, .45]	[.02, .28]	[.18, .42]	[.13, .38]	[.17, .41]	[.93, .96]	[.37, .57]	
10. CCAS	83.37	13.75	.06	.19**	.05	.17*	.10	.15*	.54**	.25**	.58**
			[-.07, .19]	[.06, .31]	[-.08, .18]	[.04, .29]	[-.03, .23]	[.02, .28]	[.44, .63]	[.12, .37]	[.49, .67]

Note. *M* and *SD* are used to represent mean and standard deviation, respectively. Values in square brackets indicate the 95% confidence interval for each correlation. The confidence interval is a plausible range of population correlations that could have caused the sample correlation (Cumming, 2014). * indicates p < .05. ** indicates p < .01.

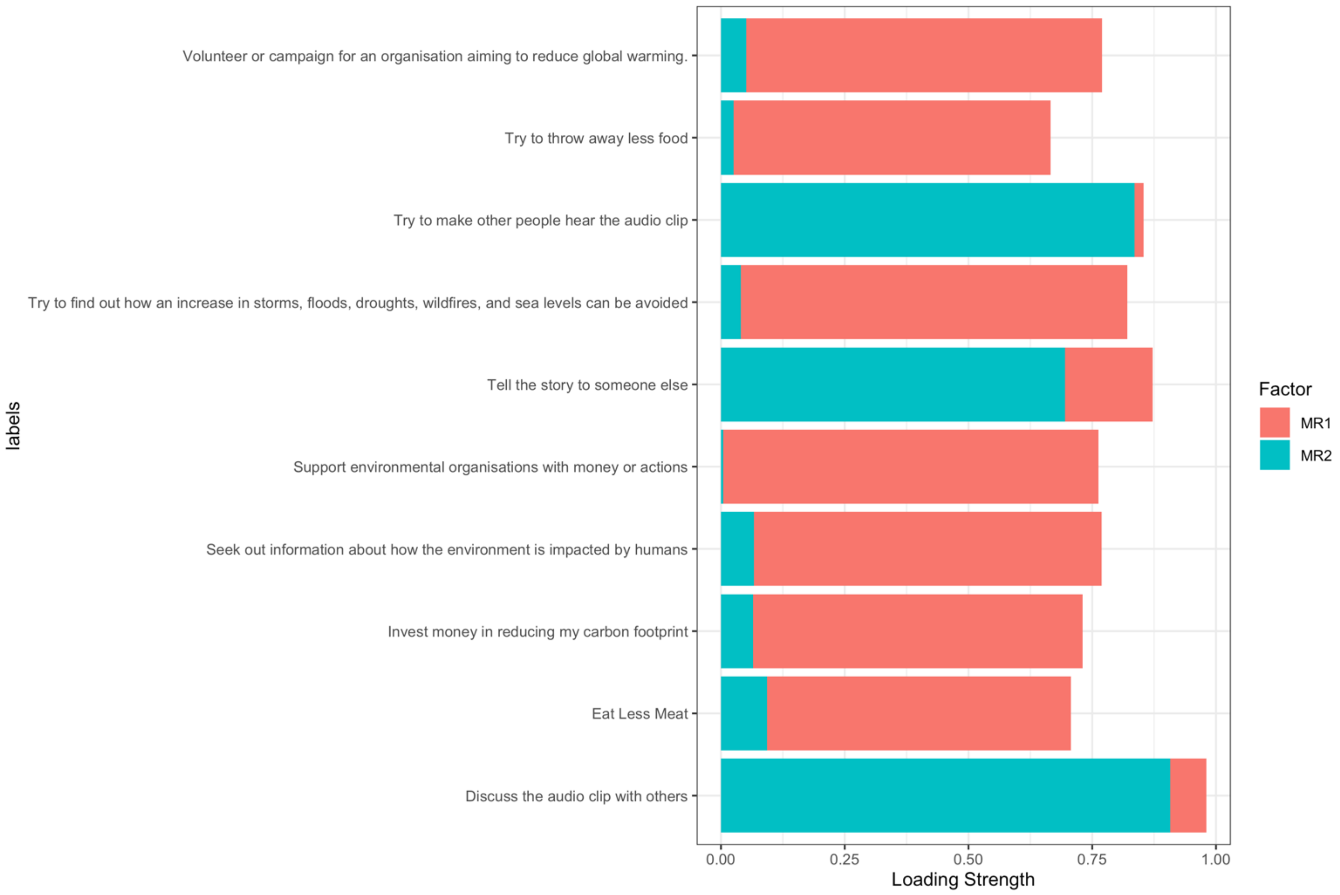

Figure S2. Overview of factor loadings of the pro-environmental intention items in Study 3.

Table S10

Regression results using General Intention and Intention to Share in Study 3

.

Predictor	*b*	*b* 95% CI [LL, UL]	*beta*	*beta* 95% CI [LL, UL]	*r*	Fit
General Intention						
(Intercept)	-20.21	[-45.71, 5.29]				
CCAS	0.39*	[0.08, 0.69]	0.19	[0.04, 0.34]	.26**	
KM	8.74**	[4.99, 12.50]	0.46	[0.26, 0.65]	.38**	
Anger	2.59	[-2.77, 7.95]	0.11	[-0.11, 0.32]	.17*	
Sadness	-2.24	[-7.43, 2.96]	-0.12	[-0.41, 0.16]	.16	
Fear	-2.58	[-7.22, 2.06]	-0.13	[-0.38, 0.11]	.13	
						R^2 = .201**<br>95% CI[.08,.29]
Intention Share						
(Intercept)	-38.92**	[-58.30, -19.53]				
CCAS	0.93**	[0.70, 1.16]	0.53	[0.40, 0.66]	.58**	
KM	2.26	[-0.60, 5.11]	0.14	[-0.04, 0.31]	.29**	
Anger	2.58	[-1.49, 6.66]	0.12	[-0.07, 0.31]	.29**	
Sadness	0.35	[-3.60, 4.30]	0.02	[-0.23, 0.27]	.27**	
Fear	-0.03	[-3.55, 3.50]	-0.00	[-0.21, 0.21]	.22**	
						R^2 = .388**<br>95% CI[.25,.47]

Note. A significant *b*-weight indicates the beta-weight and semi-partial correlation are also significant. *b* represents unstandardized regression weights. *beta* indicates the standardized regression weights. sr^2 represents the semi-partial correlation squared. *r* represents the zero-order correlation. *LL* and *UL* indicate the lower and upper limits of a confidence interval, respectively.
* indicates $p < .05$. ** indicates $p < .01$.

Results on attitudes towards climate refugees

We exploratorily tested the prediction by kama muta on attitudes toward the topic of climate refugees. We repeated the same model as earlier with the climate refugees intention variable as the DV, and kama muta, anger, fear, sadness, and prior climate attitudes as predictors. In this model we only observed that prior climate attitudes showed a significant positive prediction (β = .59 [.48, .70]). However, when inspecting the effect of kama muta on its own we found a positive association, *r* = .19 [.06, .32].

Study 4

Table S11

Means, standard deviations, and correlations with confidence intervals in Study 4.

Variable	*M*	*SD*	1	2	3	4	5	6	7	8	9	10	11	12	13
1. Condition (0 neutral, 1: moving)	0.48	0.50													
2. Kama Muta	2.06	1.39	.44**<br>[.35, .52]												
3. Communal Sharing	1.78	1.75	.48**<br>[.40, .55]	.75**<br>[.70, .79]											
4. Anger	1.71	1.59	-.22**<br>[-.31, -.12]	.19**<br>[.09, .28]	.05<br>[-.05, .15]										
5. Fear	2.18	1.63	-.25**<br>[-.34, -.15]	.31**<br>[.21, .39]	.11*<br>[.01, .20]	.68**<br>[.62, .73]									
6. Sadness	2.29	1.56	-.33**<br>[-.42, -.24]	.17**<br>[.08, .27]	-.01<br>[-.11, .09]	.71**<br>[.66, .76]	.79**<br>[.76, .83]								
7. Hope	2.99	1.69	.46**<br>[.38, .53]	.71**<br>[.66, .76]	.62**<br>[.56, .68]	-.09<br>[-.18, .01]	.04<br>[-.06, .14]	-.11*<br>[-.20, -.01]							
8. Intentions	45.88	27.20	.02<br>[-.08, .12]	.52**<br>[.45, .59]	.40**<br>[.31, .48]	.36**<br>[.27, .44]	.48**<br>[.40, .55]	.39**<br>[.30, .47]	.43**<br>[.34, .50]						
9. Intentions General	48.39	27.23	.00<br>[-.10, .10]	.47**<br>[.39, .55]	.35**<br>[.26, .44]	.34**<br>[.24, .42]	.46**<br>[.38, .54]	.38**<br>[.29, .46]	.40**<br>[.31, .48]	.97**<br>[.97, .98]					
10. Intentions Share	40.06	32.40	.06<br>[-.04, .16]	.54**<br>[.46, .60]	.43**<br>[.34, .51]	.35**<br>[.26, .43]	.43**<br>[.35, .51]	.34**<br>[.25, .43]	.40**<br>[.31, .48]	.89**<br>[.87, .91]	.76**<br>[.71, .80]				
11. Donations	0.38	0.40	-.02<br>[-.12, .08]	.10<br>[-.00, .20]	.01<br>[-.09, .11]	.11*<br>[.01, .21]	.17**<br>[.07, .27]	.13*<br>[.03, .22]	.16**<br>[.06, .26]	.33**<br>[.23, .41]	.33**<br>[.24, .41]	.27**<br>[.17, .36]			
12. Reading time	2.62	1.02	.06<br>[-.04, .16]	-.01<br>[-.11, .09]	-.08<br>[-.18, .02]	-.10<br>[-.20, .00]	-.02<br>[-.12, .08]	-.02<br>[-.12, .08]	.11*<br>[.01, .21]	.09<br>[-.01, .19]	.08<br>[-.02, .18]	.09<br>[-.01, .18]	.35**<br>[.26, .43]		
13. CCAS	4.15	0.67	-.01<br>[-.11, .09]	.18**<br>[.08, .28]	.06<br>[-.04, .16]	.15**<br>[.05, .25]	.30**<br>[.21, .39]	.27**<br>[.17, .36]	.25**<br>[.15, .34]	.49**<br>[.41, .56]	.52**<br>[.45, .59]	.35**<br>[.26, .43]	.26**<br>[.16, .35]	.11*<br>[.01, .21]	
14. EC	3.98	0.78	-.04<br>[-.18, .10]	.17*<br>[.03, .30]	.07<br>[-.07, .21]	.04<br>[-.10, .18]	.17*<br>[.03, .30]	.15*<br>[.01, .29]	.19**<br>[.05, .32]	.29**<br>[.15, .41]	.31**<br>[.18, .43]	.20**<br>[.06, .33]	.09<br>[-.06, .23]	.04<br>[-.10, .18]	.41**<br>[.28, .52]

Note. M and *SD* are used to represent mean and standard deviation, respectively. Values in square brackets indicate the 95% confidence interval for each correlation. The confidence interval is a plausible range of population correlations that could have caused the sample correlation (Cumming, 2014). * indicates $p < .05$. ** indicates $p < .01$.

Table S12

Regression results using General Intention and Intention to Share in Study 4.

Predictor	*b*	*b* 95% CI [LL, UL]	*beta*	*beta* 95% CI [LL, UL]	*r*	Fit
General Intentions						
(Intercept)	-40.29**	[-52.78, -27.80]				
Climate Attitude	14.52**	[11.28, 17.75]	0.36	[0.28, 0.44]	.52**	
Kama Muta	3.91**	[1.67, 6.15]	0.20	[0.08, 0.31]	.47**	
Anger	1.63	[-0.22, 3.47]	0.10	[-0.01, 0.20]	.34**	
Sadness	1.31	[-1.01, 3.62]	0.07	[-0.06, 0.21]	.38**	
Fear	2.70*	[0.54, 4.86]	0.16	[0.03, 0.29]	.46**	
Hope	2.93**	[1.09, 4.78]	0.18	[0.07, 0.30]	.40**	
						R^2 = .489** 95% CI[.41,.54]
Intention to Share						
(Intercept)	-32.74**	[-48.57, -16.90]				
Climate Attitude	8.80**	[4.70, 12.90]	0.18	[0.10, 0.27]	.35**	
Kama Muta	7.74**	[4.90, 10.58]	0.33	[0.21, 0.45]	.54**	
Anger	3.07*	[0.73, 5.41]	0.15	[0.04, 0.27]	.35**	
Sadness	0.45	[-2.49, 3.38]	0.02	[-0.12, 0.16]	.34**	
Fear	3.06*	[0.32, 5.80]	0.15	[0.02, 0.29]	.43**	
Hope	2.48*	[0.15, 4.82]	0.13	[0.01, 0.25]	.40**	
						R^2 = .420** 95% CI[.34,.48]

Note. A significant *b*-weight indicates the beta-weight and semi-partial correlation are also significant. *b* represents unstandardized regression weights. *beta* indicates the standardized regression weights. sr^2 represents the semi-partial correlation squared. *r* represents the zero-order correlation. *LL* and *UL* indicate the lower and upper limits of a confidence interval, respectively.
* indicates $p < .05$. ** indicates $p < .01$.

Table S13

Regression results using Donation Behavior and Reading Time as the criterion excluding hope.

Predictor	*b*	*b* 95% CI [LL, UL]	*beta*	*beta* 95% CI [LL, UL]	*r*	Fit
Donation Behavior						
(Intercept)	-0.24	[-0.48, 0.0$]				
Climate Attitudes	0.13**	[0.07, 0.20]	0.22	[0.12, 0.33]	.26**	
KM	0.01	[-0.02, 0.04]	0.02	[-0.08, 0.13]	.10	
Anger	0.01	[-0.03, 0.04]	0.03	[-0.11, 0.18]	.11**	
Sadness	-0.01	[-0.06, 0.03]	-0.06	[-0.23, 0.12]	.13**	
Fear	0.03	[-0.01, 0.07]	0.12	[-0.06, 0.29]	.17**	
						R^2 = .077** 95% CI[.02,.12]
Reading Time (log)						
(Intercept)	1.97**	[1.33, 2.62]				
Climate Attitudes	0.18*	[0.02, 0.34]	0.12	[0.01, 0.22]	.11*	
KM	-0.01	[-0.09, 0.07]	-0.02	[-0.12, 0.09]	-.01	
Anger	-0.11*	[-0.20, -0.01]	-0.16	[-0.31, -0.02]	-.10	
Sadness	0.02	[-0.10, 0.14]	0.03	[-0.15, 0.22]	-.02	
Fear	0.02	[-0.09, 0.13]	0.04	[-0.14, 0.22]	-.02	
						R^2 = .029 95% CI[.00,.06]

Note. A significant *b*-weight indicates the beta-weight and semi-partial correlation are also significant. *b* represents unstandardized regression weights. *beta* indicates the standardized regression weights. sr^2 represents the semi-partial correlation squared. *r* represents the zero-order correlation. *LL* and *UL* indicate the lower and upper limits of a confidence interval, respectively.
* indicates $p < .05$. ** indicates $p < .01$.

Analyses with Empathic Concern

We observed a positive correlation between kama muta ratings and trait empathic concern (*r* = .17 [.03, .30]), as well as between empathic concern and pro-environmental intentions (*r* = .29 [.15, .41]). We did not observe statistically significant correlations between trait empathic concern and donation behavior (*r* = .09 [-.06, .23]) or time spent on the donation page (*r* = .04 [-.10, .18]). We observed a statistically significant interaction between kama muta ratings and trait empathic concern on pro-environmental intentions. The moderating impact of trait empathic concern was smaller for higher ratings of kama muta. We did not observe statistically significant interactions for donation behavior or time spent.

Table S14.

Overview of influence of empathic concern (EC) and kama muta (KM) on intentions, donation behavior, and time spent in Study 4.

			**Intentions**			
Predictors	*Estimates*	*std. Beta*	*CI*	*standardized CI*	*p*	*std. p*
(Intercept)	-21.67	0.02	-47.66 – 4.31	-0.10 – 0.13	0.102	0.758
EC	12.23	0.19	5.64 – 18.82	0.07 – 0.30	**<0.001**	**0.002**
KM	20.47	0.54	10.00 – 30.94	0.42 – 0.65	**<0.001**	**<0.001**
EC * KM	-2.60	-0.11	-5.17 – -0.03	-0.21 – -0.00	**0.047**	**0.047**
Observations	193					
R^2 / R^2 adjusted	0.376 / 0.366					

			**Donation**			
Predictors	*Estimates*	*std. Beta*	*CI*	*standardized CI*	*p*	*std. p*
(Intercept)	-0.12	0.02	-0.58 – 0.35	-0.13 – 0.16	0.621	0.802
EC	0.12	0.07	-0.00 – 0.23	-0.08 – 0.21	0.057	0.361
KM	0.16	0.04	-0.03 – 0.35	-0.10 – 0.19	0.097	0.555
EC * KM	-0.04	-0.11	-0.08 – 0.01	-0.24 – 0.03	0.115	0.115
Observations	192					
R^2 / R^2 adjusted	0.022 / 0.007					

			**Time Spent**			
Predictors	*Estimates*	*std. Beta*	*CI*	*standardized CI*	*p*	*std. p*
(Intercept)	2.01	0.01	0.77 – 3.26	-0.13 – 0.15	**0.002**	0.879
EC	0.19	0.04	-0.12 – 0.51	-0.10 – 0.19	0.227	0.545
KM	0.18	-0.08	-0.32 – 0.69	-0.23 – 0.06	0.471	0.259
EC * KM	-0.06	-0.07	-0.18 – 0.06	-0.20 – 0.07	0.328	0.328
Observations	193					
R^2 / R^2 adjusted	0.013 / -0.002					

Note: EC = Empathic concern; KM = kama muta

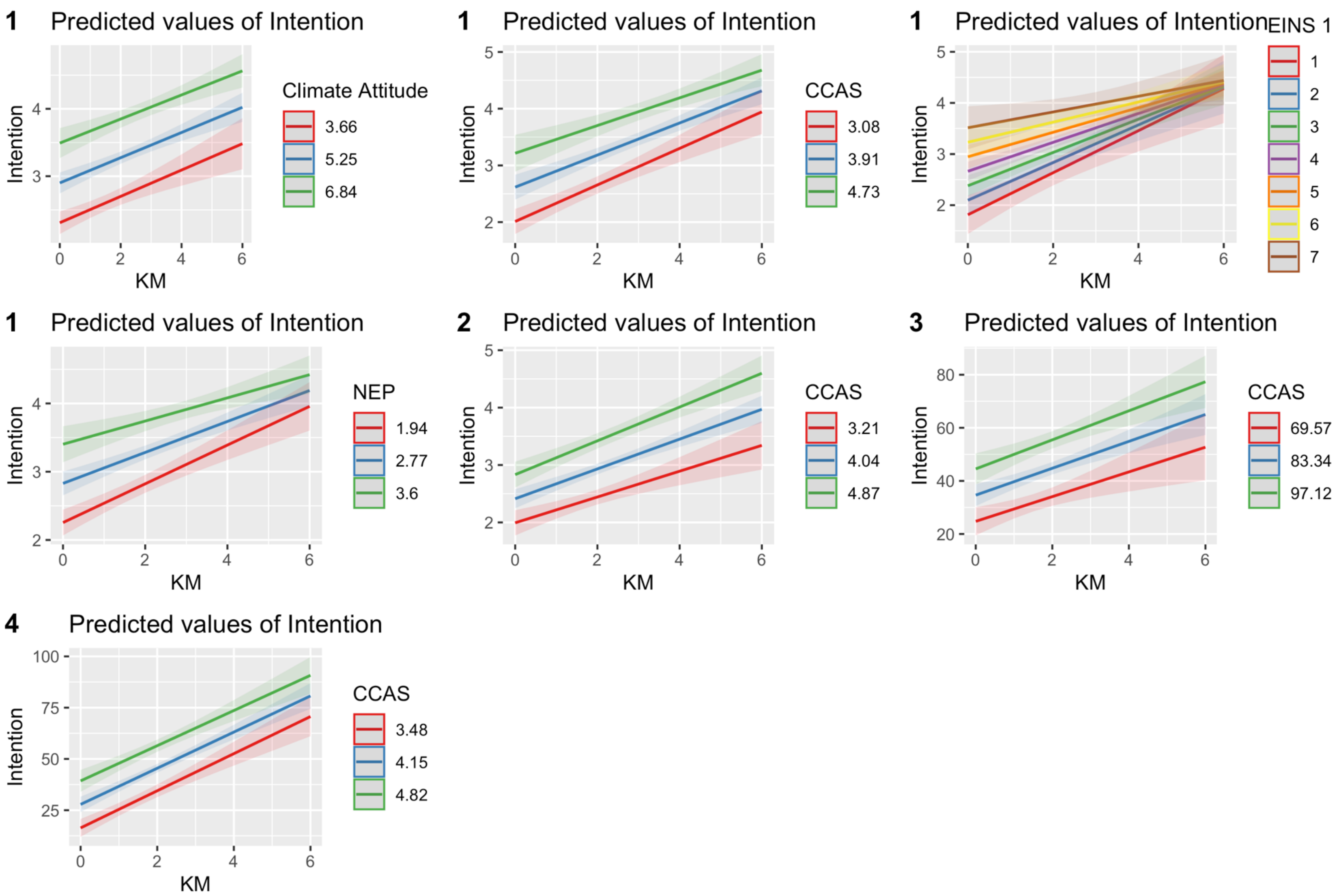

Figure S3. Overview of moderation models of climate attitudes on the relationship between kama muta and pro-environmental intentions across Studies 1-4. CCAS = Climate Change Attitude Survey, EINS = Inclusion of the Environment in the Self, NEP = New Ecological Paradigm.

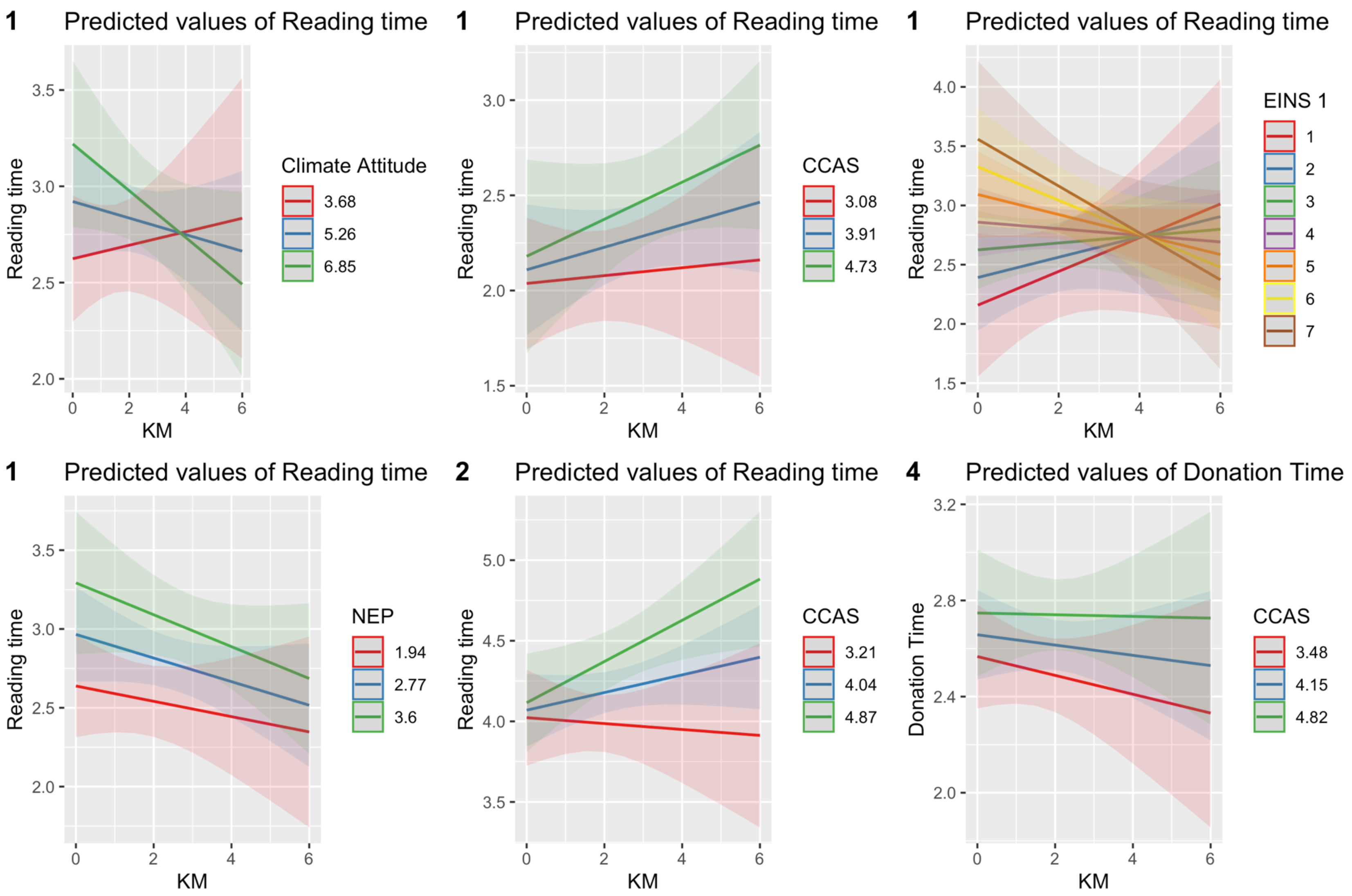

Figure S4. Overview of moderation models of climate attitudes on the relationship between kama muta and reading time across Studies 1-2 & 4.

Mediation Models

Table S15

Overview of mediation models including path a, b, and c' and the direct, indirect, and total effects across Study 2-4.

	Estimate	SE	*z*	*p*
**Study 2**				
Condition -> Kama Muta -> Intentions				
Intention				
Condition	-0.34	0.11	-3.14	.002
Kama Muta	0.35	0.03	9.95	<.001
Kama Muta				
Condition	0.80	0.20	3.91	<.001
direct	-0.34	0.11	-3.14	.002
indirect	0.28	0.08	3.60	<.001
total	-0.06	0.13	-0.50	.620
Condition -> Kama Muta -> Reading Time				
Reading Time				
Condition	-0.13	0.13	-1.03	.304
KM	0.10	0.04	2.42	.016
KM				
Condition	0.80	0.20	3.90	<.001
direct	-0.13	0.13	-1.03	.304
indirect	0.08	0.04	1.89	.059
total	-0.05	0.13	-0.43	.666
Condition -> Communal Sharing -> Intentions				
Intention				
Condition	-0.32	0.13	-2.56	.011
CS	0.22	0.03	6.84	<.001
CS				
Condition	1.15	0.22	5.26	<.001
direct	-0.32	0.13	-2.56	.011
indirect	0.26	0.06	4.19	<.001
total	-0.06	0.13	-0.49	.621
**Study 3**				
Condition -> Kama Muta -> Pro Environ. Intentions				
Intention				
Condition	-6.59	2.92	-2.25	.024
KM	7.82	1.20	6.50	<.001
KM				
Condition	1.24	0.16	7.71	<.001
direct	-6.59	2.92	-2.25	.024
indirect	9.72	1.92	5.05	<.001
total	3.14	2.96	1.06	.288

	**Estimate**	**SE**	***z***	***p***
**<u>Study 4</u>**				
Condition -> Kama Muta -> Pro Environ. Intentions				
<u>Intention</u>				
Condition	-13.90	2.42	-5.74	<.001
KM	12.49	0.78	16.08	<.001
<u>KM</u>				
Condition	1.21	0.13	9.53	<.001
direct	-13.90	2.42	-5.74	<.001
indirect	15.15	1.90	7.97	<.001
total	1.25	2.75	0.45	.650
Condition -> Kama Muta -> Donation				
<u>Donation</u>				
Condition	-0.06	0.04	-1.44	.149
KM	0.04	0.02	2.39	.017
<u>KM</u>				
Condition	1.20	0.13	9.34	<.001
direct	-0.06	0.04	-1.44	.149
indirect	0.05	0.02	2.28	.023
total	-0.02	0.04	-0.43	.668
Condition -> Kama Muta -> Time Spent				
<u>Time Spent</u>				
Condition	0.16	0.12	1.42	.155
KM	-0.03	0.04	-0.79	.432
<u>KM</u>				
Condition	1.21	0.13	9.42	<.001
direct	0.16	0.12	1.42	.155
indirect	-0.04	0.05	-0.78	.435
total	0.13	0.11	1.19	.234
Condition -> Communal Sharing -> Pro Environ. Intentions				
<u>Intention</u>				
Condition	-11.97	2.80	-4.27	<.001
CS	7.87	0.77	10.25	<.001
<u>CS</u>				
Condition	1.68	0.16	10.78	<.001
direct	-11.97	2.80	-4.27	<.001
indirect	13.22	1.86	7.09	<.001
total	1.25	2.76	0.45	.650
Condition -> Communal Sharing -> Donation				
<u>Donation</u>				
Condition	-0.03	0.05	-0.57	.569
CS	0.00	0.01	0.38	.705
<u>CS</u>				
Condition	1.68	0.16	10.56	<.000
direct	-0.03	0.05	-0.57	.569
indirect	0.01	0.02	0.37	.708
total	-0.02	0.04	-0.43	.668

	**Estimate**	**SE**	***z***	***p***
Condition -> Communal Sharing -> Time Spent				
Time Spent				
Condition	0.27	0.12	2.31	.021
CS	-0.09	0.03	-2.56	.011
CS				
Condition	1.68	0.16	10.79	<.001
direct	0.27	0.12	2.31	.021
indirect	-0.14	0.06	-2.50	.012
total	0.13	0.10	1.22	.223
Condition -> Felt Manipulation -> Pro Environ. Intention				
Intention				
Condition	0.98	2.65	0.37	.710
Felt Manipulated	-3.77	0.89	-4.22	<.001
Felt Manipulated				
Condition	-0.07	0.17	-0.41	.680
direct	0.98	2.65	0.37	.710
indirect	0.27	0.66	0.40	.688
total	1.25	2.73	0.46	.647
Condition -> Felt Manipulation -> Donation				
Donation				
Condition	-0.02	0.04	-0.48	.631
Felt Manipulation	-0.04	0.01	-3.26	.001
Felt Manipulation				
Condition	-0.06	0.17	-0.34	.737
direct	-0.02	0.04	-0.48	.631
indirect	0.00	0.01	0.32	.748
total	-0.02	0.04	-0.42	.672
Condition -> Felt Manipulation -> Time Spent				
Time Spent				
Condition	0.12	0.10	1.17	.240
Felt Manipulation	-0.06	0.03	-2.18	.029
Felt Manipulation				
Condition	-0.07	0.17	-0.41	.685
direct	0.12	0.10	1.17	.240
indirect	0.00	0.01	0.36	.721
total	0.13	0.10	1.21	.225

Note: Predictor -> Mediator -> Outcome.
Underscored is the criterion for the current model, underneath are the predictors in that model.

Sensitivity Power Analyses

We performed a sensitivity analysis using G*Power 3.0 (Faul et al., 2007) in order to investigate what effect size we were able to detect at 95% power (alpha = .05, one-tailed) given our final sample sizes (Figure S5). For studies 1-3 we were able to detect effect sizes as small as *r* = .22 with 95% power (*r* = .17 with 80% power). In Study 4, we were able to detect effect sizes as small as *r* = .17 with 95% power (*r* = .13 with 80%). Thus, given our sensitivity analyses our studies were powered enough to obtain the *stronger* effect with pro-environmental intentions, but they were less adequate powered to optimally detect *smaller* effects as the relationship with actual behavior.

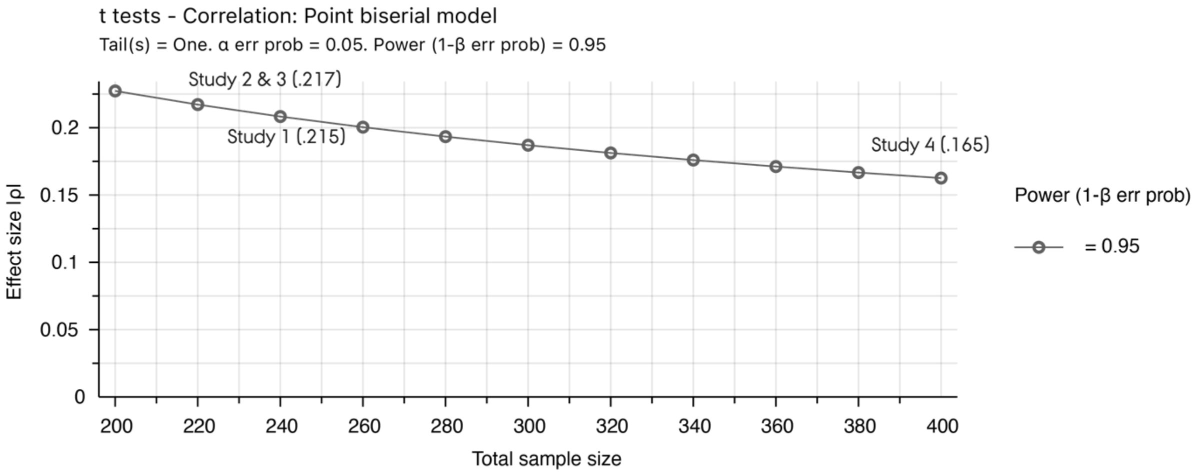

Figure S5.
Sensitivity plot of effect size by total sample size for a power of 95% (alpha .05, one tailed).

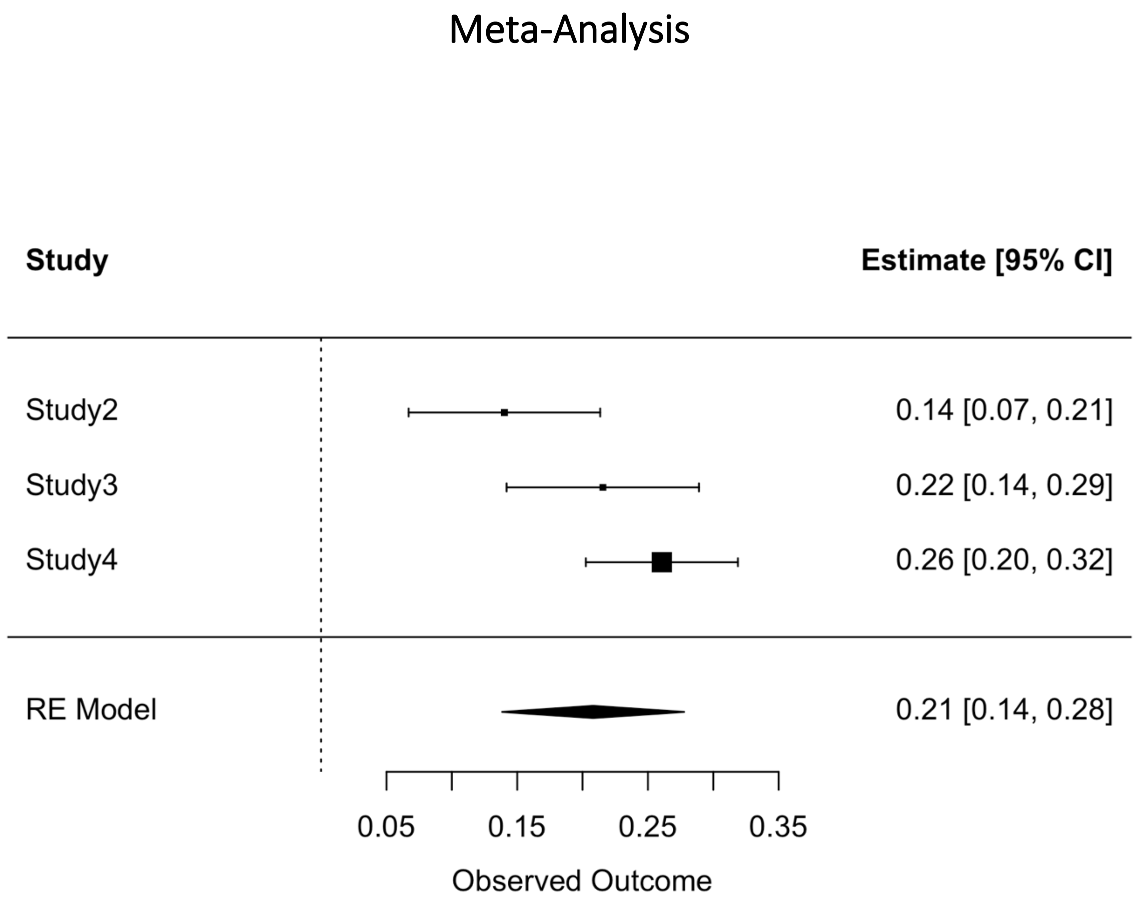

Figure S6.
Forest plot of indirect effect of kama muta on the relation between the experimental manipulation and intentions. $Q(2) = 6.37$, $p = .041$, $I^2 = 68.13$ [0, 99.18].

Stimuli

Study 1

Man vs. Earth: https://www.youtube.com/watch?v=VrzbRZn5Ed4
Future: https://www.youtube.com/embed/7Rglp-CMKW8

Study 2

Control: https://www.youtube.com/watch?v=ZO6cfqDA6eM (from 1:00 to 2:30 only)
Kama Muta: https://www.youtube.com/watch?v=PTA6oewQVqE

Study 3

Control: https://www.youtube.com/watch?v=LcICDeWutSw
Kama Muta: https://www.youtube.com/watch?v=ZO6cfqDA6eM (full clip)

Study 4

Kama Muta (Future): https://www.youtube.com/embed/7Rglp-CMKW8
Control: https://www.youtube.com/watch?v=m9T247StoQo

References

Firke, S. (2021). Janitor: Simple tools for examining and cleaning dirty data. *R package version 2.1.0.*

Hayes, A. F. (2017). *Introduction to mediation, moderation, and conditional process analysis: A regression-based approach*. Guilford publications.

Kassambara, A., & Kassambara, M. A. (2020). Package 'ggpubr'. *R package version 0.4.0*

Lüdecke, D. (2018). sjPlot: Data visualization for statistics in social science. *R package version 2.8.11*

Revelle, W. R. (2017). psych: Procedures for personality and psychological research.

Rosseel, Y. (2012). lavaan: An R package for structural equation modeling. *Journal of Statistical Software*, *48*, 1-36.

Stanley, D. J., & Spence, J. R. (2018). Reproducible tables in psychology using the apaTables package. *Advances in Methods and Practices in Psychological Science*, *1*(3), 415-431.

Viechtbauer, W. (2010). Conducting meta-analyses in R with the metafor package. *Journal of Statistical Software*, *36*(3), 1–48.

Wickham, H. (2016). Data analysis. In *ggplot2* (pp. 189-201). Springer, Cham.

Wickham, H. (2017). reshape2: flexibly reshape data: a reboot of the reshape package. *R package version 1.4.4*

Wickham, H., Averick, M., Bryan, J., Chang, W., McGowan, L. D. A., François, R., ... & Yutani, H. (2019). Welcome to the Tidyverse. *Journal of open source software*, *4*(43), 1686.

Wilke, C. O. (2019). cowplot: streamlined plot theme and plot annotations for "ggplot2". *R package version 3.3.6*
